# Investigation of Chemical Constituents and Antioxidant Activity of Biologically Active Plant-Derived Natural Products

**DOI:** 10.3390/molecules28145572

**Published:** 2023-07-21

**Authors:** Katarzyna Godlewska, Paweł Pacyga, Agnieszka Najda, Izabela Michalak

**Affiliations:** 1 Department of Pharmacology and Toxicology, The Faculty of Veterinary Medicine, Wrocław University of Environmental and Life Sciences, 50-375 Wrocław, Poland; 2 Department of Thermodynamics and Renewable Energy Sources, Faculty of Mechanical and Power Engineering, Wrocław University of Science and Technology, 50-370 Wrocław, Poland; pawel.pacyga@pwr.edu.pl; 3 Department of Vegetable and Herbal Crops, The University of Life Science in Lublin, 20-950 Lublin, Poland; agnieszka.najda@up.lublin.pl; 4 Department of Advanced Material Technologies, Faculty of Chemistry, Wrocław University of Science and Technology, 50-372 Wrocław, Poland; izabela.michalak@pwr.edu.pl

**Keywords:** plants, extraction, ultrasound-assisted extraction, natural products, rapid screening, bioactive compounds, antioxidant activity

## Abstract

The aim of this publication is to present rapid screening methods (visual/colorimetric) that will enable quick identification of the presence of biologically active compounds in aqueous solutions. For this reason, 26 plant extracts obtained by ultrasound-assisted extraction were analysed for the content of these compounds. Higher plants, used as a raw material for extraction, are common in Europe and are easily available. The article proposes a comparison of various protocols for the identification of various compounds, e.g., phenolic compounds (phenols, tannins, anthocyanins, coumarins, flavones, flavonoids), vitamin C, quinones, quinines, resins, glycosides, sugars. Initial characterisation of the composition of plant extracts using fast and inexpensive methods allows you to avoid the use of time-consuming analyses with the use of advanced research equipment. In addition, the antioxidant activity of plant extracts using spectrophotometric methods (DPPH, ABTS, FRAP assay) and quantitative analysis of plant hormones such as abscisic acid, benzoic acid, gibberellic acid, indole acetic acid, jasmonic acid, salicylic acid, zeatin, zeatin riboside, and isipentenyl adenine was performed. The obtained results prove that the applied visual methods show different sensitivity in detecting the sought chemical compounds. Therefore, it is necessary to confirm the presence or absence of bioactive substances and their concentration using modern analytical methods.

## 1. Introduction

Plants were used as a primary raw material for medical therapies until the invention of synthetic drugs in the 19th century [1,2,3,4,5]. Plants are a notable source of natural chemicals, with various structural and biological features that exhibit multifarious mechanisms of action [6,7,8]. The various plant species contain myriad secondary metabolites (substances produced by cells through the metabolic pathway) that greatly influence their competitiveness in the environment and protection against adverse growth conditions [7,9,10,11]. These substances are also known to exhibit a great value for humans [8,12]. The plant-based bioactive compounds can be classified according to biological pathways and chemical classes, among which main chemical groups can be distinguished, such as: alkaloids, furanocoumarins, glycosides (anthraquinone glycosides, cardiac glycosides, cyanogenic glycosides, glucosinolates, and saponins), lignans, naphthodianthrones, peptides, phenolic compounds (anthocyanins, flavonoids, hydroxycinnamic and phenolic acids, and stilbenes), phenylpropanoids, proteins, tannins (condensed tannins—polymers of flavonoids, hydrolysable tannins—polymers of a monosaccharide core with several catechin derivatives attached), mono-, di-, and sesquiterpenoids, and resins [2,6,9,11,12,13,14,15]. In particular, the development of natural products containing substances isolated from natural origin has increased in recent years due to their high efficacy, safety, and long-term health effects [3,5,12,16,17,18,19]. They have been applied in many fields, including beverages, cosmetics, dyeing, flavouring, fragrances, medicine (e.g., steroids and alkaloids), nutrition and functional foods (e.g., sterols and stanols as cholesterol-lowering ingredients), repellents, smoking, and other industrial purposes [1,8,10,11,12,18,20]. However, to source these valuable components, which can occur in small quantities, it is crucial to employ the appropriate extraction, purification, and separation methods [7,8]. Generally, isolation is carried out in accordance with widely recognised techniques concerning complete extraction (e.g., maceration, steam- or hydro-distillation, pressing, boiling, infusion, percolation, Soxhlet extraction, microwave-assisted extraction (MAE), ultrasound-assisted extraction (UAE), accelerated solvent extraction (ASE), supercritical fluid extraction (SFE), pressurised fluid extraction (PFE), enzyme-assisted extraction (EAE), subcritical water extraction (SWE), ionic liquid extraction (ILE), pulse electric field extraction (PEFE)), preferably with nontoxic solvents (e.g., water, carbon dioxide, ethanol, ionic liquids) [7,8,10,11,12,19,20,21,22]. The biomass extract preparation includes general pre-treatment (e.g., liquid-liquid extraction, solid-phase extraction, gel filtration) and pre-concentration (e.g., gel filtration, solid-phase extraction, molecularly imprinted polymers, microporous absorption resin) [7,23,24]. Depending on the intended application of the obtained extracts, the biological assays (e.g., antibacterial, antifungal) can also be performed [7,25,26]. The activity-oriented separation (off-line: e.g., preparative scale bioguided fractionation, HPLC micro-fractionation; on-line: e.g., HPLC post-column (bio)chemical detection, biochromatography, electrophoretic enzyme assays) could also be considered. The final step to obtain phytocomplex or single molecules is the structure elucidation by means of off-line methods (e.g., UV-DAD, MS, NMR) or hyphenated techniques (e.g., HPLC-UV-DAD, HPLC-MS, GC-MS, HPLC-SPE-NMR, UPLC-DAD-TOF-MS) [7,27,28,29]. The technical and economic viability of any extraction and purification process should be evaluated in order to select production processes, marketing strategies, and remunerativeness [19].

The impressive contribution of plant-based extracts to virtually all aspects of human life has promoted their use to an increasing extent. For this reason, it is crucial to accelerate and reduce the cost of the production of new and innovative bioproducts and solutions. In view of the fact that the extraction process is a crucial first step in the development of new formulations, the research within this article has been designed to present the methods that could be used as a primary screening when no data are available on the chemical composition of examined extracts to evaluate the efficiency of the extraction techniques, to ensure that the active ingredients were not destroyed during preparation, and thus to reduce the time and costs of further purification of the obtained natural products. The choice of our examined plants was based on the ease and economic acquisition of raw materials (plants commonly found in the natural environment) and richness of active compounds that may be found in them. A total of 26 different extracts were tested for the content of phenolic compounds (phenols, tannins, anthocyanins, coumarins, flavones, flavonoids), vitamin C, quinones, quinines, resins, glycosides, sugars, antioxidant activity, and plant hormones. In most of the analyses, basic qualitative methods were used to provide a quick answer regarding the content of specific active compounds. The extracts were produced by means of ultrasound-assisted extraction, which is considered as a more environmentally friendly technique while allowing the extraction of bioactive compounds on a larger scale.

The aim of the publication was a comprehensive characterisation of plant extracts obtained from a number of higher plants. A given compound was determined using a series of methods, due to which it was possible to select visual protocols, the most sensitive ones indicating the presence of the given compound in the extract.

## 2. Results

The tested methods allowed rapid identification of the presence or absence of bioactives in the extract; however, in order to determine the exact amount of tested compounds, it is necessary to use more sophisticated analytical methods.

Throughout the paper, the following abbreviations were used for the particular extract: Alv L (solution/extract prepared based on aloe leaves), Am Fr (black chokeberry fruits), Arv H (common mugwort herb), Bv R (beetroot roots), Co F (common marigold flowers), Ea H (field horsetail herb), Ep F (purple coneflower flowers), Ep L (purple coneflower leaves), Hp H (St. John’s wort herb), Hr Fr (sea-buckthorn fruits), Lc S (red lentil seeds), Mc F (chamomile flowers), Ob H (basil herb), Pm H (broadleaf plantain herb), Poa H (common knotgrass herb), Ps S (pea seeds), Pta L (common bracken leaves), Sg L (giant goldenrod leaves), So R (comfrey roots), To F (common dandelion flowers), To L (common dandelion leaves), To R (common dandelion roots), Tp F (red clover flowers), Ur L (nettle leaves), Ur R (nettle roots), Vo R (valerian roots). In order to better visualise the obtained effects, the tables also show the tube with the extract before treatment (always the first on the left). The changes were usually observable immediately (up to 5 min) after following the appropriate procedures.

### 2.1. Phenolic Compounds (Total Phenolic Compounds, Tannins, Anthocyanins, Coumarins, Flavones, Flavonoids)

Several protocols for rapid phytochemical screening can be used to determine the presence of bioactive compounds in the examined samples. According to the literature, to assess the prevalence of phenolic compounds the ferric chloride test can be implemented. Authors who used this method found that these compounds were present after the appearance of a dark green [30,31,32], deep blue [31], violet [33], bluish black [34,35,36], or bluish-green [36] colour. Similar results were presented by other researchers who stated that violet [37], blue or green [38], or deep blue or black colour [39] indicates the presence of phenols. The second method, the lead acetate test, is also widely applied to detect these compounds in samples. Their presence can be confirmed when white precipitate is developed [31,40]. However, it is worth mentioning that the lead acetate test reveals very little helpful information and has the drawback of involving the heavy metal, lead, which creates environmental disposal problems. As a third method, the zinc hydrochloride test can be deployed—the appearance of yellow or orange colour after a few minutes proves the presence of phenols [37]. In another method, the Shinoda test, a yellow or orange colour demonstrates their existence [37]. The total phenolic content can be quantified using the Folin–Ciocalteu test, and when the bluish colour occurs it confirms the presence of phenolic compounds and their concentrations are verified by measuring the absorbance of the solutions [41].

The formation of green-blue [39], violet or blackish red [33,37] colouration in the ferric chloride test [39]; the yellow precipitate in the lead acetate test [31,37,39,42,43]; a red or magenta colour in the zinc hydrochloride test [37]; or a pink scarlet, green to blue, or crimson red colour emerging within minutes in the Shinoda test indicates flavonoids [31,33,36,37,43]. In the alkaline reagent test, the addition of sodium hydroxide solution causes an intense yellow colour which changes to colourless after the addition of hydrochloric acid, which may also suggest their presence [30,34,37]. In the aluminium chloride test, if the addition of aluminium chloride solution induces the light-yellow colour, the existence of flavonoid is observed. The addition of sodium hydroxide and hydrochloric acid makes the solution colourless, which also confirms their presence [32,39]. Among other methods used to identify these compounds, the ammonium test (a yellow colour at the ammonia layer [37,39]), the ammonia and sulphuric acid test (a yellow colour [30]), and the Millon’s test (a white precipitate which turns to red after gentle heating [37]) can be mentioned. Photos of the Millon’s test are presented in our previous article, where we conducted the analyses of proteins [44].

The ferric chloride test is likewise used for the analysis of tannins. Their presence can be confirmed when the formation of a greenish black precipitate [38,39,40,42,43,45] or a green, violet [37], or dark blue [33,38] colour is observed. Other authors have stated that the greater addition of ferric chloride changes the blue or greenish black colour to olive green [46]. The occurrence of a blackish blue colour indicates the presence of gallic tannins and a green-blackish colour shows the presence of catechol tannins [47]. The yellow [34,37,45,48] or white coloured precipitate in the lead acetate test [42,43] or a yellow to red precipitate in the alkaline reagent test may indicate the presence of tannins in the solution [37]. These compounds can also be detected in samples using other tests, among others: gelatin test (the white precipitate [37]), potassium dichromate test (the yellowish brown colour precipitate) [45], HCl test (the red coloured precipitate—phlobatannins [34,38,39,47]), and bromine water test (the buff coloured precipitate—condensed tannins; no precipitate—hydrolysable tannins [49]).

The sodium hydroxide test is employed in the analysis of anthocyanins (a blue-green colour [39]) and coumarins and flavones (a yellow colour [33,34,38,42]). In the sulphuric acid test, the yellowish orange colour indicates flavones [40] or anthocyanins, orange to crimson indicates flavonones, and yellow to orange colour indicates flavones [37].

The bromine water test can be used to detect the presence of glycosides (a yellow precipitate develops [37]) and carbohydrates (the solution discolours (by aldose)) [37]).

In the case of our results, the ferric chloride test clearly identified the presence of phenolic compounds in the following extracts: Am Fr, Ea H, Ep F, Ep L, Hr Fr, Pm H, Poa H, To F, To L, Tp F, Ur L. The colour of the extracts changed into a uniform dark green colour, without precipitation or turbidity of the solution (Table 1). Quantitative analysis of total polyphenol content, with the use of the Folin–Ciocalteu test, confirmed that qualitative methods show different sensitivities—indicating the presence of TPC in extracts that contain a great as well as a low amount of them but do not show their content even although these compounds are present. The highest levels of TPC could be found in Ep F, Pta L, and Ep L (3.2–2.2 mg·mL^−1^) and the lowest in Ps S, Ur R, Ur L, Lc S, and To R (0.07–0.18 mg·mL^−1^) (Table 1). The appearance of a white precipitate in the lead acetate test indicates the presence of phenols—Table 2. This was observed with the following extracts: Alv L, Bv R, Mc F, Ob H, Pm H, Ur R. White precipitation can also indicate the presence of tannins. Evident yellow/orange colour of the solution, which is typical for the presence of phenols in the zinc hydrochloride test, was observed in the extract Bv R, Co F, Ep F, Ep L, Sg L, Tp F, Ur L, Ur R, Vo R (Table 2). The results for the Shinoda test in most cases coincide with the results for the zinc hydrochloride test, used to detect phenols in plant extracts (Table 2).

The ferric chloride test was inconclusive in the determination of flavonoids in the extracts (Table 1). According to literature data, the appearance of a green-blue colour may indicate the presence of flavonoids [39]. No such change was observed for any of the tested extracts. The potential presence of flavonoids in the extract should be confirmed by another method. The Millon’s test (vide Table 4 in the work of Godlewska et al. [44]) revealed that the formation of precipitation, which could be considered as a positive result for the presence of flavonoids, occurred only in tubes with Co F (before boiling (1) an orange-brown precipitation formed, while after boiling (2) a white precipitation formed), EP F ((1) a brown precipitate, (2) a red precipitate), Lc S ((1) a white precipitate, (2) a white precipitate), Ps S ((1) a yellowish precipitate, (2) a white precipitate), So R ((1) a brown precipitate, (2) a brick-red precipitate). Yellow precipitation in the lead acetate test typical to tannins and flavonoids present in extracts was detected for Arv H, Co F, Ea H, Poa H, Pta L, To F, To L, To R, Vo R (Table 2). The colour change of the extract in the zinc hydrochloride test to red/magenta, indicating the presence of flavonoids, performed with the same test, was observed only for the extract Am Fr (Table 2). The presence of flavonoids detected by the Shinoda test was in the following extracts: Am Fr, Bv R, Ep F, Hr Fr, Lc S, Ps S, Tp F. The Shinoda test was more effective in detecting flavonoids in plant extracts than the zinc hydrochloride test (Table 2). The use of the alkaline reagent test did not allow the detection of flavonoids in plant extracts (Table 3). The ammonium test did not give a clear answer as to the content of flavonoids (Table 6). The unequivocal yellow colour, which indicates the presence of flavonoids in plant extracts, was observed only for Hr Fr, Poa H, and To R. A yellow colour, which indicates the presence of flavonoids in extracts using the ammonia and H_2_SO_4_ test, was observed for Co F, Ea H, Hr Fr, Mc F, To F, To R, and Tp F. After applying the ammonium chloride test, discoloration of the solution to some degree could be observed in most cases (with the exception of Am Fr).

In the case of tannin identification using the ferric chloride test, in addition to the greenish-black colour, which is typical for phenolic compounds, a precipitate was also observed, especially in the following extracts: Arv H, Bv R, Hp H, Mc F, Ob H, Pta L, Sg L, So R, Vo R (Table 1). In the case of the determination of tannins by the gelatin test, a change in the colour of the extract was mainly observed, and not the formation of a characteristic white precipitate (Table 3). This has been seen with the following extracts: Hp H, Lc S, and Ps S. A yellow to red precipitate indicating the presence of tannins in the plant extracts (alkaline reagent test) was present only in a few extracts: Ea H, Lc S, Pta L, To F, and Ur L (Table 3). Using the bromine water test, no tannins were detected in most botanical extracts (Table 3). The use of the potassium dichromate test did not allow the detection of tannins in plant extracts (Table 4). For this reason, the dichromate test for identifying tannins is not recommended, as it has given all negative results, and additionally dichromate poses a disposal issues. Furthermore, the bromine water test very rarely gave positive outcome for any class of compound and could easily be recommended not to be used. The characteristic yellowish-brown precipitate was not observed. A similar situation occurred in the case of detecting tannins (phlobatannins) with the HCl test. Dark (red) colour precipitate was observed only in the following extracts: So R and Vo R (Table 4).

Using the NaOH test, the presence of anthocyanins was not detected in the botanical extracts (Table 5). In none of the cases was the colour of the extract blue-green. The appearance of a yellow colour in the extract during this test indicates the presence of coumarins and flavones. Such a colour was unequivocally observed in the extracts Hr Fr, Mc F, Poa H, and To R. The H_2_SO_4_ test in many cases did not give a clear answer as to the presence of anthocyanins and flavones in plant extracts. A stable yellowish-orange colour that indicated the presence of flavones and anthocyanins was observed for Alv L, Am Fr, Hp H, Poa H, Pta L, Tp F, Ur L.

### 2.2. Vitamin C

In the DNPH test (2,4-dinitrophenylhydrazine), the formation of yellow precipitate indicates the presence of vitamin C [34]. The presence of vitamin C, using the DNPH test, was observed only for Lc S and Ps S extracts (Table 6).

### 2.3. Quinones, Quinines, Resin

The literature shows that the sulphuric acid test (the appearance of red colour) [38,42,43], the hydrochloric acid test (the formation of yellow precipitation) [34,37], and the ammonia test (a pink coloured precipitate) [38] can be applied to detect the presence of quinones/anthraquinones. In the sodium hydroxide test, a deep colouration (e.g., purple, red) can be attributed to the presence of quinine [33]. Furthermore, in the acetone test, a turbid solution implies the presence of resin [33].

The application of the H_2_SO_4_ test, HCl test, ammonia test, and NaOH test did not allow the detection of quinones and quinines in plant extracts (Table 7).

The acetone test was used to detect resins in plant extracts. Their presence (turbidity of the solution) was confirmed in the following extracts: Alv L, Am Fr, Arv H, Co F, Ea H, Lc S, Ob H, Pm H, Ps S, Pta L, Sg L, So R, To F, Ur R, and Vo R.

### 2.4. Glycosides

Glycosides can be found in samples using a number of rapid approaches. Authors who used the Keller–Killiani test showed that the presence of brown [36,38,42,43] or a reddish-brown ring at the junction of two layers [45] indicates the appearance of cardiac glycosides. Other authors stated that cardiac glycosides are present in sample when the colour of the acidic layer above the ring changes to bluish green [37,45] or greenish [36] and the lower layer to reddish brown [37] or violet [36]. In the Baljet test, the yellow to orange colour exhibits the occurrence of cardiac glycosides [37]. In the Borntrager’s tests (1), the anthraquinone glycosides can be found in samples when the ammoniacal (lower) layer shows a rose, pink, or red colour [37,39,42,43,50]. In the modified Borntrager’s tests (2), the pink colour indicates the presence of glycosides [32,38]. In the sulphuric acid test, the appearance of reddish precipitate indicates the presence of glycosides [40]. Photos are available in our previous article, in analyses of protein content (vide Table 4 in the work of Godlewska et al. [44]). The Molisch test can also be used as another method. In this protocol, the formation of a reddish-violet ring at the junction of two layers confirms the presence of glycosides [40]. The next method is Liebermann’s test, in which the appearance of a colour from violet through blue to green suggests the presence of glycosides [34]. Photos are presented in our previous article (vide Table 5 in the work of Godlewska et al. [44]).

No glycosides were detected in most botanical extracts using the bromine water test (Table 3). The use of the Baljet test did not show the presence of cardiac glycosides in most of the extracts tested (Table 8). Molisch’s test can be used to quickly screen extracts for the content of glycosides and sugars. The appearance of a reddish-violet ring at the junction of two liquids was easily visible in many botanical extracts (Table 9). The Borntrager test (2) was not effective in the detection of glycosides as well as sugars, and neither was the Borntrager test (1) in the detection of cyanogenic glycosides in the tested plant extracts. In all tubes subjected to the Liebermann’s test, no violet or blue colour was observed, which could likewise indicate the presence of these compounds. Extracts that may be considered to contain glycosides to some extent due to the greenish colour are Ep L and Mc F.

The Keller–Killiani test (Table 9), like the Baljet test (Table 8), did not provide full clarity on the presence of cardiac glycosides in plant extracts.

### 2.5. Sugars

Various protocols can be used to detect the presence of sugars. One of them is the Fehling’s test. The simple (reducing) sugars are present in samples when first a yellow, then a brick red precipitate is noted [31,33,37,39,42,43,45,47]. The next one is Benedict’s test—when the solution turns green [42,43] or red [31,40], or if the reddish-brown precipitate forms [33] it might suggest the presence of carbohydrates/reducing sugars. In the Molisch’s test, the appearance of a purple or reddish colour [38,47] or purple [30,34,37,40,45] or red brown [31,40,45] coloured ring at the junction of the two liquids shows the occurrence of carbohydrates. Additionally, the Borntrager’s test can also be applied, and when a change in colour of the ammonia layer is observed it indicates the presence of carbohydrates [37]. In the Selwinoff’s test, a red colouration implies fructose content in the solution [37], while in the Barfoed’s test, the formation of red precipitation reveals the presence of monosaccharaides [47].

The deployment of the bromine water test did not allow the determination of sugars in most botanical extracts (Table 3). No yellow/red precipitate was observed after using Fehling’s test, indicating the presence of sugars in the extracts (Table 10). Benedict’s test showed a clear change in the colour of the extract to green and the formation of a red-brown precipitate, which indicated the presence of sugars (reducing sugars) in almost all extracts tested. Selwinoff’s test gives a red coloured compound when linked with resorcinol. The colour of the extracts changed to red for Am Fr, Bv R, and Hp H. The red precipitate is the result of the Barfoed test, which indicates the presence of simple sugars and was observed in the following extracts: Arv H, Pta L, and To R.

### 2.6. Antioxidant Activity

Plant-derived extracts possessed varied antioxidant activity (Table 11). The analysis conducted using the DPPH assay showed that the highest radical scavenging potential demonstrated the following extracts: Pta L, Hp H, Ep F, Am Fr, Sg L, To L, and Ob H (9.57–2.48 µM Trolox·mL^−1^) and the lowest: Lc S, Ur L, Ur R, and Ps S (0.14–0.15 µM Trolox·mL^−1^). The greatest DPPH inhibition ratio showed extracts based on Pm H, Hr Fr, and Arv H (31.58–28.12%), while the smallest were based on Lc S, Ur L, Ps S, Ur R, and Ep L (2.00–2.37%). On the other hand, the relative ability of the antioxidants present in bioproducts to scavenge the ABTS free radicals was the strongest in Ep L, Ep F, Hp H, Am Fr, To L, Poa H, and Pta L (19.00–6.33 µM Trolox·mL^−1^), and the weakest in To R, Alv L, Ur L, Ea H, Hr Fr, and Lc S (0.81–1.90 µM Trolox·mL^−1^). The ABTS inhibition ratio was the highest for Poa H (5.37%) and So R (4.47%) and the lowest for Ob H, Sg L, and Pta L (0.34–0.54%). The most effective scavenging of the FRAP radical exhibited compounds present in extracts Pta L, Ep L, Ep F, Ob H, Sg L, Hp H, and Am Fr (20.25–8.73 µM Trolox·mL^−1^), while the least were in Lc S, Ps S, Ur R, To R, Ur L, and Alv L (0.40–1.38 µM Trolox·mL^−1^).

### 2.7. Plant Hormones

Of the seven plant hormones analysed (Table 12), gibberellic acid (GA_3_) was present in extracts in the highest amounts, especially in Sg L, Ur R, Pm H, To R, Ur L, and Ep F (359–319 μg∙mL^−1^). The following bioproducts, Arv H, Pta L, Hr Fr, Hp H, and Tp F (29.07–76.90 μg∙mL^−1^), contained the lowest amounts of GA_3_. The indole acetic acid (IAA) occurred in high levels in Ps S, Pm H, Ep F, To R, and Hr Fr (2.71–1.93 μg∙mL^−1^), while there were trace amounts in Arv H and Pta L. However, Arv H along with Hp H, Ob H, Ep F, Tp F, and Mc F (1.0–1.5 μg∙mL^−1^) contained the highest quantity of abscisic acid (ABA), whereas the amount of ABA in Am Fr, Ea H, Hr Fr, Lc S, Poa H, Ps S, Pta L, Ur L, and Ur R was at levels below detection. The concentration of benzoic acid (BA) was the highest in To R, To L, Ob H, and Pm H (0.48–0.28 μg∙mL^−1^), while it was present in trace amounts in Co F, Ea H, Hp H, Hr Fr, Poa H, Sg L, Ur L, and Ur R. Jasmonic acid (JA), salicylic acid (SA), and zeatin (Z) were present in trace amounts in most extracts. The quantity of SA was the highest in Lc S, Ea H, and Poa H (0.15–0.11 μg∙mL^−1^), while Z was highest in To F, So R, and To L (21.0–17.0 μg∙mL^−1^).

## 3. Discussion

Phenolic compounds (PCs) have well-documented beneficial effects on human health and exhibit antioxidant, anti-inflammatory, antimicrobial, antiviral, antitumoral, antidiabetic, anti-obesity, antiallergic, anti-lipidemic, antiproliferative, neuroprotective, and cardioprotective activities [51,52]. The main PCs include phenolic acids, flavonoids (flavonols, flavones, flavanones, flavanols, isoflavonoids, anthocyanins), tannins, stilbenes, and lignans [51,52,53,54]. These compounds are used in various industries, including food, nutraceutical, cosmetic, packaging, textile, pharmacy, and medicine [52,55,56,57].

Among the rapid, qualitative methods used to assess the presence of phenolic compounds can be mentioned ferric chloride test, lead acetate test, zinc hydrochloride test, Shinoda test, gelatin test, alkaline reagent test, bromine water test, potassium dichromate test, HCl test, NaOH test, H_2_SO_4_ test, aluminium chloride test, ammonium test, ammonia and H_2_SO_4_ test. By comparing these results with quantitative analysis data obtained with the use of the Folin–Ciocalteu test, it can be noted that qualitative tests vary significantly in sensitivity in detecting the targeted bioactive compounds. This assay is widely used to assess TPC in foods; however, it is not specific for their determinations and is highly dependent on the composition of the matrix, which can vary in terms of the types phenolics and the amount of particular compounds. For instance, reducing sugars or vitamin C may hamper the accuracy of this assay [58,59]. The Folin–Ciocalteu test showed that all extracts contained phenolic compounds in the range of 0.07 mg·mL^−1^ (Ps S) to 3.17 mg·mL^−1^ (Ep F). It can also be seen that the extracts prepared from Lc S and Ps S contained one of the lowest TPC contents despite the content of the vitamin C (the content of reducing sugars was not found). In contrast, the content of reducing sugars in extracts containing the highest amount of TPC, namely Ep L and Pta L, was confirmed in only one or two cases, respectively (the presence of vitamin C was not found). The point-biserial Correlation results for the comparison of methods used to detect phenolic compounds (PC) are included in Table S1. The analysis takes into account quantitative variable (Folin–Ciocalteu test results) and nominal variable (presence and absence of PC marked by plus or minus sign). There are two cases considered, depending on how to define the “−/+” sign: (a) treated as “+” (rpb+), (b) as “−“ (rpb−). The values of the point-biserial correlation coefficient rpb+ show that there is a positive, medium strength correlation for the Ferric chloride test, and a positive, low strength correlation for the Zinc hydrochloride test. When the rpb− coefficient is investigated, the findings indicate a similar pattern, with the difference that the Shinoda test is characterised by a positive, low strength correlation.

The ferric chloride test allowed detection of the presence of PC only in four extracts (Ep F, Ep L, To L, Am Fr) out of nine, with the highest concentration ranging from 3.17 mg·mL^−1^ to 1.0 mg·mL^−1^. Meanwhile, this test confirmed their presence in extracts that contained lower levels of them; for example, Ur L (0.13 mg·mL^−1^), Poa H (0.36 mg·mL^−1^), and Ea H (0.42 mg·mL^−1^). This assay was also appropriate for the determination of tannins in the following extracts: Arv H, Bv R, Hp H, Mc F, Ob H, Pta L, Sg L, So R, andVo R, but was ambiguous in the determination of flavonoids. The Acetate test allowed detection of phenols in Alv L, Bv R, Mc F, Ob H, Pm H, andUr R, as well as tannins and flavonoids in Arv H, Co F, Ea H, Poa H, Pta L, To F, To L, To R, and Vo R. The zinc hydrochloride test confirmed the presence of phenols in Bv R, Co F, Ep F, Ep L, Sg L, Tp F, Ur L, Ur R, and Vo R, and flavonoids in Am Fr. The results of the presence of phenols with the use of the Shinoda test in most cases coincide with the results for the zinc hydrochloride test, while the presence of flavonoids was verified in Am Fr, Bv R, Ep F, Hr Fr, Lc S, Ps S, and Tp F. However, the alkaline reagent test did not detect flavonoids in plant extracts. The Millon’s test can also be used to determine flavonoids, and in our extracts they were detected in Co F, EP F, Lc S, Ps S, and So R. The presence of tannins can be indicated using the gelatin test (positive for Hp H, Lc S, and Ps S), the alkaline reagent test (positive for Ea H, Lc S, Pta L, To F, and Ur L), and the HCl test (phlobatannins) (positive for So R and Vo R). However, the use of the bromine water test and the potassium dichromate test did not allow the detection of these compounds.

The NaOH test did not prove to be effective in the determination of anthocyanins, but it enabled the identification of coumarins and flavones (positive for Hr Fr, Mc F, Poa H, and To R). The H_2_SO_4_ test in many cases did not give a clear answer as to the presence of anthocyanins and flavones in plant extracts (positive for Alv L, Am Fr, Hp H, Poa H, Pta L, Tp F, and Ur L). Comparing both NaOH and H_2_SO_4_ tests for detecting anthocyanins and flavones, the latter seems to be more sensitive, but the presence of these active compounds in plant extracts was confirmed in most cases by both tests. The ammonium test did not give a clear answer as to the content of flavonoids (positive for Hr Fr, Poa H, and To R). The ammonia and H_2_SO_4_ test seems to be more precise in the detection of flavonoids in plant extracts than the ammonium test. The ammonia and H_2_SO_4_ test indicated the presence of flavonoids in Co F, Ea H, Hr Fr, Mc F, To F, To R, and Tp F. The ammonium chloride test showed that most extracts contained flavonoids (with the exception of Am Fr). The comparison of sensitivity of applied methods for the detection of polyphenolic compounds has been included in Supplementary Materials (Tables S1–S4). Among the examined tests for the presence of phenolic compounds in plant extracts, the most sensitive test was the ferric chloride test. The visual results largely coincide with the total polyphenol content, determined by the Folin–Ciocalteu test (Table S1). Failure to detect phenolic compounds with the ferric chloride test coincided with a very low concentration of these compounds in the extract using the spectrophotometric technique (Folin–Ciocalteu reagent). Phenolic compounds are common in plants and are easily extracted using water as a solvent. Based on the studies carried out, the ferric chloride test can also be recommended for the detection of tannins in plant extracts (Table S2). For the detection of flavonoids in plant extracts, many tests (aluminium chloride test, ammonium test, ammonia and H_2_SO_4_ test) gave inconclusive results. To the greatest extent, the results obtained for these tests coincided with the detection of flavonoids using the lead acetate test, which can be used as the first to screen plant extracts for the presence of flavonoids (Table S3). In the case of detecting anthocyanins in plant extracts, the NaOH test turned out to be useless—these compounds were not detected in any of the extracts tested. However, for their initial detection in extracts, the H_2_SO_4_ test can be used. The same applies to the screening of extracts for the presence of flavones. The H_2_SO_4_ test was more sensitive than the NaOH test (Table S4).

Vitamin C, an omnipresent plant and animal metabolite [60], exhibits multifarious biological and pharmaceutical functions [61]. It is crucial in the prevention of scurvy [60]; helps to lower blood cholesterol [62]; and is necessary for collagen, carnitine, and neurotransmitters biosynthesis [63,64]. It supports detoxification, assists the adequate function of the immune system, and is involved in the primary prevention of commonly encountered diseases, including diabetes, eye diseases, atherosclerosis [63] cardiovascular disease, and cancer [60]. In view of the fact that this vitamin is not synthesized by the human body, it has to be provided with diet [62]. Vitamin C is extensively utilised in the feed, food, and pharmaceutical industry as a nutritional supplement and preservative [61,65]. In our analysis, the DNPH test allowed detection of its presence only in Lc S and Ps S extracts. The study of this molecule is greatly handicapped by its oxidation under exposure to air, light, and heat.

Quinine, a cinchona alkaloid, belongs to the aryl amino alcohol group of drugs. It has played an invaluable role in the treatment of malaria since the 18th century and still plays a key role in the treatment of this disease. In turn, quinones, a class of compounds containing a benzene ring with a carbonyl group [66], are used in industry as oxidants, dehydrating agents [67], and dyes [68]. The analysis using the H_2_SO_4_ test, HCl test, ammonia test, and NaOH test did not confirm the presence of the tested compounds in any of the obtained extracts. The comparison of methods used to detect of quinones are presented in Supplementary Materials (Table S5). Among the tests for the detection of quinones in plant extracts (H_2_SO_4_ test, HCl test, ammonia test), the HCl test was the most sensitive.

Plant resins are a complex mixture of specialised metabolites; for example, alkaloids, phenols, and terpenes [69,70,71,72] as well as alcohols, aldehydes, esters, and amorphous neutral substances [69]. Due to their diverse biological activities (e.g., antimicrobial, anti-inflammatory, antioxidant, anticancer, antiulcer, haemostatic, immunostimulant) [70,72,73,74,75,76], resins are used as a raw material in the medical and pharmaceutical industry [70,73] but also as fuel additives, paint thinners, rosin, and varnishes as well as components in polishes [69]. One of the fast tests to verify the presence of resins is the Acetone test. This assay confirmed their existence in the following extracts: Alv L, Am Fr, Arv H, Co F, Ea H, Lc S, Ob H, Pm H, Ps S, Pta L, Sg L, So R, To F, Ur R, and Vo R.

Another group of compounds examined as a part of this study were glycosides, which can be sourced from plant or animal origin [77,78]. Various types of glycosides can be distinguished: among others, triterpene, β-sitosterol, flavonoid, iridoid, phenylpropanoid, anthraquinone, kaempferol, and saponin. The biological activity is strongly related to their stereochemistry [77,79]. Glycosides have been recognized and utilised as alternative drugs in the treatment of various cancers and have other notable therapeutic potential and clinical utility [77,79,80]. For instance, flavonoid glycosides possess antioxidant, anti-inflammatory, anti-allergic, anti-microbial, and anti-cancer activities and thus find use in the prevention and management of diseases [78,79]. Cardiac glycosides are used for the treatment of cardiac arrhythmia, congestive heart failure, and atrial fibrillation; exhibit strong anticancer activity; and evoke cell proliferation or activation of cell death by apoptosis or autophagy [77,78,81,82]. Visualisation of the presence of glycosides can be conducted with the use of various methods. The Molisch’s test proved to be the most sensitive in detecting these compounds (positive for Alv L, Arv H, Co F, Ea H, Lc S, Mc F, Ob H, Poa H, Ps S, Sg L, So R, Tp F, Ur L, and Ur R). However, the Borntrager test (1), the Borntrager test (2), the Keller–Killiani test, the Baljet test, and the bromine water test did not provide reliable confirmation of the presence of glycosides in plant extracts. The use of the Liebermann’s test also did not assure a full clarity of their appearance. The extracts which could be to some extent considered as a glycoside containing are Ep L and Mc F. The summary of protocols used for the confirmation of the presence of glycosides can be found in Supplementary Materials (Table S6). For the detection of glycosides in plant extracts, Molisch’s test is undoubtedly recommended.

The principal source of sugars, the main products of photosynthesis [83,84,85], are beet and cane sugar, while other sources may include honey, corn syrup, fruits, and vegetables [86]. The most abundant free sugars found in plants are disaccharides (sucrose and maltose) and monosaccharides (glucose and fructose) [83,87]. These compounds are used in food products to provide sweetness and energy, but also play a key role in preservation, fermentation, colour, flavour, and texture [86,88,89]. The highest sensitivity in determining the presence of sugars showed the Benedict’s test (all extracts with the exception of Lc S and Ps S) and Molisch’s test (positive for Alv L, Arv H, Co F, Ea H, Lc S, Mc F, Ob H, Poa H, Ps S, Sg L, So R, Tp F, Ur L, and Ur R). Selwinoff’s test (positive for Am Fr, Bv R, and Hp H) and the Barfoed test (positive for Arv H, Pta L, and To R) proved to be less effective in the identification of carbohydrates. The use of Fehling’s test, the Borntrager test (2), and the bromine water test were not sensitive in the detection of sugars. The comparison of methods used for the detection of sugars has been included in Supplementary Materials (Table S7). Both Molisch’s test and Benedict’s test were effective in detecting sugars in the tested plant extracts.

Antioxidants, compounds able to prevent/inhibit/reduce oxidation processes [90,91], can be sourced from microorganisms, plants, and animal tissues [92]. The industry has utilised them to prevent metal corrosion and oxidative degradation of polymers (e.g., rubbers, plastics, and adhesives), but they have also found use as food preservatives (enrichment and inhibition of disruption, sourness, and colour change) [90,91,92,93], and as stabilisers in fuels and lubricants [91,93], but also in pharmacology, cosmetics, and medicine [92] (in the prevention of degenerative illnesses, e.g., cancers, cardiovascular, and neurological diseases, cataracts and oxidative stress dysfunctions) [93]. In recent years, due to their numerous biological activities (e.g., anti-aging and anti-inflammatory), the interest in the utilisation of antioxidants is rapidly growing [92]. The measurements of antioxidant activity with the use of three examined assays (DPPH, ABTS, and FRAP assays) revealed that Pta L, Hp H, Ep F, and Am Fr had the highest reducing power. Additionally, the greatest antioxidant activity was also noted for Sg L, Ob H (DPPH assay and FRAP assay), To L (DPPH assay and ABTS assay), and Ep L (ABTS assay and FRAP assay). The extract Poa H was characterised by one of the highest activities in the ABTS test, while in the DPPH test and FRAP test it was characterised by one of the lowest. The lowest reducing power was observed for Vo R, Ea H, Poa H, To R, Alv L, Ur R, Ps S, Ur L, and Lc S (all three assays) as well as for Ur R and Ps S (DPPH assay and FRAP assay). Therefore, it can be seen that despite the differences between these tests, the results obtained are relatively comparable.

Plant hormones, which can be found in plants, algae, and plant-associated bacteria and fungi, play a vital role in plant growth and development (e.g., promote fruit ripening and leaf drop, stimulate seed germination and gemmation, increase yield and resistance to adverse environmental conditions) [94,95,96,97]. The use of these compounds in agriculture and horticulture is of great importance, and since their first discovery and commercial availability, farmers have incorporated them into the crop production to improve numerous aspects of the cultivation processes [96,98,99]. The conducted studies proved that the obtained extracts could constitute a source of plant hormones, especially gibberellic acid (e.g., Ep F, Pm H, Sg L, To R, Ur L, Ur R).

## 4. Materials and Methods

### 4.1. Chemicals and Reagents

The following chemicals were used in this study: sodium carbonate (Sigma Aldrich, St. Louis, MI, USA), sodium hydroxide (Avantor, Radnor Township, PA, USA), sulphuric acid (Avantor), ammonium hydroxide (Supelco, Bellefonte, PA, USA), acetone (Stanlab, Lagos, Nigeria), chloroform (Avantor), acetic acid (Supelco), glacial acetic acid (Supelco), hydrochloric acid (Avantor), iron chloride (Sigma Aldrich), lead acetate (Sigma Aldrich), zinc dust (Roth), magnesium turnings (Sigma Aldrich), Folin–Ciocalteu’s phenol reagent (Sigma Aldrich), sodium carbonate (Sigma Aldrich), gelatin (Sigma Aldrich), sodium chloride (Sigma Aldrich), bromine water (Carlo Erba, Milan, Italy), potassium dichromate (Sigma Aldrich), aluminium chloride (Sigma Aldrich), 2,4-dinitrophenylhydrazine (PanReac AppliChem, Darmstadt, Germany), sodium picrate (Merck, Rahway, NJ, USA), Trolox (Sigma Aldrich), gallic acid (Sigma Aldrich), diphenyl−2-picrylhydrazyl (DPPH) (Sigma Aldrich), azino-bis−3-ethylbenzthiazoline6-sulphonic acid (ABTS) (Sigma Aldrich), tripyridyl-S-triazine (TPTZ) (Sigma Aldrich), ethanol (TH.GEYER, Höxter, Germany), methanol (TH.GEYER), mercuric nitrate (Sigma Aldrich), mercurous nitrate (Alfa Aesar, Haverhill, MA, USA), nitric acid (Merck), ammonia solution 25% (Supelco), α-naphthol (Carlo Erba), copper (II) sulphate (Sigma Aldrich), potassium tartrate (Sigma Aldrich), trisodium citrate dihydrate (Alfa Aesar), resorcinol (Sigma Aldrich), copper acetate (Roth), phytohormone standards Z, BA, JA, SA, ABA (Sigma-Aldrich), GA_3_, IAA (OlChemIm Ltd., Olomouc, Czech Republic), methanol (HPLC quality, Merck), acetonitrile (HPLC quality, Merck), and acetic acid (HPLC quality, Merck).

### 4.2. Plant Materials Used for the Production of Extracts

The main factors in the selection of raw materials were their prevalence in Europe, ease, and low cost of acquisition, as well as the content of biologically active compounds [44]. The biomasses were purchased (FLOS, Herbisarium) or collected from the natural environment (Wrocław, Poland). The harvesting time was adjusted to the level of biologically active components in the plants (based on literature data). The list of plants (with abbreviations) being used, included aloe leaves, black chokeberry fruits, common mugwort herb, beetroot roots, common marigold flowers, field horsetail herb, purple coneflower flowers, purple coneflower leaves, St. John’s wort herb, sea-buckthorn fruits, red lentil seeds, chamomile flowers, basil herb, broadleaf plantain herb, common knotgrass herb, pea seeds, common bracken leaves, giant goldenrod leaves, comfrey roots, common dandelion flowers, common dandelion leaves, common dandelion roots, red clover flowers, nettle leaves, nettle roots, and valerian roots.

### 4.3. Extraction

Plant-based extracts were produced through ultrasound-assisted extraction (UAE) with the use of a UP 50 H homogeniser (Hielscher Ultrasonics GmbH, Brandenburg, Germany). Raw materials (dried, 500 μm mesh size) were macerated with deionised water (ratio 1:20 *w*/*v*) at room temperature. After 30 min, the mixtures were sonicated (30 min) and centrifuged (4500 rpm, 10 min, Heraeus Megafuge 40, rotor TX-750, Thermo Scientific, Waltham, MA, USA). The analyses of bioactive compounds and antioxidant activity were performed in the obtained supernatants [44].

### 4.4. Analyses of Extracts

#### 4.4.1. Phenolic Compounds

##### Total Phenolic Compounds

Ferric chloride test—to each extract (3 mL), neutral ferric chloride solution (5%, 5 drops) was added [30].

Lead acetate test—to each extract (2.5 mL), a lead acetate solution (10%, 1.5 mL) was added [40].

Zinc hydrochloride test—to each extract (3 mL), a pinch of zinc dust and concentrated HCl were added (5 drops) [37].

Shinoda test—to each extract (3 mL), few turnings of magnesium and concentrated HCl (5 drops) were added [37].

Folin–Ciocalteu test—to the extracts (0.1 mL), Folin–Ciocalteu’s phenol reagent (0.2 mL) and distilled water (2.0 mL) were added, and the solution was incubated (room temperature, 3 min). Then, Na_2_CO_3_ (20 mg·mL^−1^, 1.0 mL) was added, and the mixtures were incubated in the dark (1 h). The absorbance was determined at 765 nm using a spectrophotometer (Varian Cary 50 Conc. Instrument, Victoria, Australia). The results were expressed as gallic acid equivalents (GAE) [100].

##### Tannins

Gelatin test—to the extracts (3 mL), 1% gelatin solution containing 10% sodium chloride (15 drops) was added [37].

Alkaline reagent test—to the extracts (3 mL), NaOH (20%, 10 drops) was added [37].

Bromine water test—to the extract solution (2 mL), bromine water (0.2 mL) was added [49].

Ferric chloride test—to each extract (3 mL), neutral ferric chloride solution (5%, 5 drops) was added [39].

Lead acetate test—to each extract (2.5 mL), lead acetate solution (10%, 1.5 mL) was added [45].

Potassium dichromate test—to each extract (5 mL), potassium dichromate solution (10%, 1 mL) was added [45].

HCl test (phlobatannins)—to each extract (2 mL), HCl (1%, 2 mL) was added [38], then the mixture was boiled (5 min) [34,39].

##### Anthocyanins

NaOH test—each extract was treated with NaOH (10%, 2 mL) [39].

H_2_SO_4_ test—extracts (3 mL) were treated with H_2_SO_4_ (15 drops) [37].

##### Coumarins

NaOH test—each extract was treated with NaOH (10%, 2 mL) [34,42].

##### Flavones

NaOH test—each extract was treated with NaOH (10%, 2 mL) [40].

H_2_SO_4_ test—extracts (3 mL) were treated with H_2_SO_4_ (15 drops) [37].

##### Flavonoids

Alkaline reagent test—to the extracts (3 mL), NaOH (20%, 10 drops) and HCl (20%, 10 drops) were added [37].

Aluminium chloride test—extracts (2 mL) were shaken with AlCl_3_ solution (1%, 0.5 mL). Next, NaOH (20%, 0.5 mL) and HCl (20%, 0.5 mL) were added [39].

Ammonium test—extracts (1 mL) were treated with NH_3_(aq) (10%, 2 mL) and H_2_SO_4_ (5 drops) [39].

Ammonia and H_2_SO_4_ test—to each extract (1 mL), ammonia solution (10%, 2 mL) and concentrated H_2_SO_4_ (5 drops) were added [30].

Ferric chloride test—to each extract (3 mL), neutral ferric chloride solution (5%, 5 drops) was added [39].

Lead acetate test—to each extract (2.5 mL), lead acetate solution (10%, 1.5 mL) was added [37].

Millon’s test—extracts (2 mL) were mixed with Millon’s reagent (2 mL) and boiled (5 min) (Ramya et al., 2019). Methodology similar to the methodology of proteins described in our previous article [44].

Shinoda test—to each extract (3 mL), a few turnings of magnesium and concentrated HCl (5 drops) were added [37].

Zinc hydrochloride test—to each extract (3 mL), a pinch of zinc dust and concentrated HCl were added (5 drops) [37].

#### 4.4.2. Vitamin C

DNPH test—2 mL of the test solution was treated with 2,4-dinitrophenyl hydrazine dissolved in conc. H_2_SO_4_ [34].

#### 4.4.3. Quinones

H_2_SO_4_ test—extracts (2 mL) were shaken (5 min) with conc. H_2_SO_4_ (2 mL) [43].

HCl test—extracts (2 mL) were treated with HCl (5 mL) [34].

Ammonia test (anthraquinones)—to each extract (2 mL), NH_3_(aq) (10%, 15 drops) was added [38].

#### 4.4.4. Quinines

NaOH test—extracts (1 mL) were mixed with NaOH (5%, 1 mL) [33].

#### 4.4.5. Resin

Acetone test—extracts (1 mL) were treated with acetone (1 mL) [33].

#### 4.4.6. Glycosides

Borntrager test (cardiac glycosides)—extracts (5 mL) were treated with conc. H_2_SO_4_ (1 mL), glacial acetic acid (2 mL) and FeCl_3_ solutions (5%, 3 drops) [42].

Baljet test (cardiac glycosides)—extract (2 mL) were mixed with a solution of sodium picrate (5 drops) [37].

Bromine water test (cardiac glycosides)—to the extract solution (2 mL), bromine water (0.2 mL) was added [37].

Borntrager’s tests (1) (cyanogenic glycosides)—diluted H_2_SO_4_ (2 mL) was added to each extract (2 mL). Solution was boiled (10 min) and filtered. The filtrate (1 mL) was shaken with chloroform (1 mL), then the separated chloroform layer (lower part) was shaken with NH_3_ solution (10%, 0.5%) [39].

Borntrager’s tests (2)—extracts (2 mL) were mixed with chloroform (2 mL) and NH_3_ solution (2 mL) [32,38].

H_2_SO_4_ test (glycosides)—extracts (3 mL) were treated with H_2_SO_4_ (1 mL) (Shetty and V 2012). Methodology similar to the methodology of proteins described in our previous article [44].

Molisch’s test—to each extract (2 mL), Molisch’s reagent (2 drops, ethanolic solution of α-naphthol (5%)) was added and mixed well. Next, conc. H_2_SO_4_ (1 mL) was added and allowed to stand for a few minutes [40].

Liebermann’s test—methodology similar to previously described analyses of steroids (vide: Liebermann–Burchard test for steroids, [44]). Extracts (1 mL) were mixed with chloroform (1 mL) and acetic acid (2 mL). Then, conc. H_2_SO_4_ (2 drops) was added [34].

#### 4.4.7. Sugars

Fehling’s test—Fehling’s A solution (aqueous solution of copper (II) sulphate) (1 mL) and Fehling’s B solution (solution of potassium tartrate) (1 mL) were mixed and boiled (1 min). Next, extracts (2 mL) were added to the above mixture and boiling continued (water bath, 5 min) [42].

Benedict’s test—Benedict’s reagent (2 mL) and extracts (2 mL) were mixed and heated (boiling water bath, 5 min) [42]. Benedict’s solution consisted of 17.0 g of trisodium citrate dihydrate, 10.0 g of anhydrous sodium carbonate, 1.74 g of copper (II) sulphate, and 100 mL of water.

Molisch’s test—to each extract (2 mL), Molisch’s reagent (2 drops, ethanolic solution of α-naphthol (5%)) was added and mixed well. Next, conc. H_2_SO_4_ (1 mL) was added and allowed to stand for few minutes [34].

Bromine water test—to the extract solution (2 mL) 0.2 mL of bromine water [37] was added.

Borntrager’s test—extracts (2 mL) were mixed with chloroform (2 mL) and NH_3_ solution (2 mL) [37].

Selwinoff’s test—to the extracts (3 mL), Selwinoff’s reagent (1 mL) was added and boiled (10 min) [37]. Selwinoff’s solution was prepared by dissolving 110 mg of resorcinol in 220 mL of 3N HCl.

Barfoed’s test—extracts (2 mL) were mixed with Barfoed’s reagent (1 mL) and heated (water bath, 2 min) [47]. Barfoed’s solution was prepared by dissolving 13.3 g of copper acetate in 200 mL of water and then 1.8 mL of glacial acetic acid was added.

#### 4.4.8. Antioxidant Activity

DPPH assay—extracts (0.5 mL, diluted 100 or 1000 times) were mixed with ethanol (1.5 mL) and DPPH solution (0.5 mL), vigorously shaken, and left in the dark (10 min). The absorbance was measured at 517 nm [100].

ABTS assay—extracts (30 µL) were mixed with ABTS solution (3 mL) and left in the dark (6 min). The absorbance was measured at 734 nm [100].

FRAP assay—extracts (1 mL) were mixed with FRAP solution (3 mL) and after 10 min the absorbance at 593 nm was measured [100].

The percentage of DPPH and ABTS scavenging effects were calculated by the following equation:

(1) $$Inhibition\;ratio\;(\%)=\frac{A_{control}-A_{sample}}{A_{control}}\times 100\%$$

where *A_control_* is the absorbance of the addition of ethanol and *A_sample_* is the absorbance of tested extracts.

#### 4.4.9. Plant Hormones

HPLC—qualitative and quantitative HPLC chromatographic analysis of plant hormones were performed in the reverse phase system, using a LaChrom-Merck liquid chromatograph with a DAD diode detector (L-7450), a pump (L-7100), a degasser (L-7612), a 20 µL dosing loop with a thermostat (L-7360), a Rheodyne dispenser, and a steel column LiChrocart C18 250 mm × 4.6 mm filled with a stationary phase with a grain diameter of dp = 5 µm. The samples were analysed at 30 °C. Separation of standard substances was performed using an isocratic elution in 1% aqueous solution of acetic acid and acetonitrile (75:25, *v*/*v*) at pH 4.0. Mobile phases for the determination of hormones in the plant samples consisted of 40% acetonitrile—0.1% acetic acid in water (eluent A) and 0.1% acetic acid in methanol (eluent B). The following gradient was used: 0–18 min, 100% A; 18–25 min, linear gradient up to 100% B; 25–35 min 100% B; 35–40 min, linear gradient to 100% A. Post-run time was 15 min. Elution was performed with a solvent flow rate of 0.8 mL·min^−1^ and an injection size of 20 µL. Detection was carried out at a wavelength of λ = 230 to 287 nm. Hormones were identified by comparing their retention times (tR) with the standards. Abscisic acid (ABA), benzoic acid (BA), gibberellic acid (GA3), indole acetic acid (IAA), jasmonic acid (JA), salicylic acid (SA), zeatin (Z), zeatin riboside (RZ), and isipentenyl adenine (IP) in the tested extracts was calculated on the basis of a calibration curve determined for each identified hormone. All samples were filtered through 0.22 µm membrane filters before injection into HPLC [101,102].

## 5. Conclusions

The current study represents the systematic screening of bioactive compounds extracted from twenty-six biomasses. The detailed phytochemical study of the content of phenolic compounds (phenols, tannins, anthocyanins, coumarins, flavones, flavonoids), vitamin C, quinones, quinines, resins, glycosides, and sugars, as well as antioxidant activity and the content of plant hormones, have been reported. The applied protocols are accessible, inexpensive, and provide a quick answer regarding the presence or absence of bioactive compounds. Several methods could be used for rapid screening, while modern analytical methods are necessary for the final confirmation of the concentration of bioactive compounds.

## Figures and Tables

**Table 1:** The results of ferric chloride test and Folin–Ciocalteu test (PC—phenolic compounds, TN—tannins, FD—flavonoids).

Method	Ferric Chloride Test					Folin–Ciocalteu Test
Extract	Observation	PC	TN	FD	Photo	mg·mL^−1^
**Alv L**	A change in the colour of the solution to brown with a green glow was observed	−	−	−	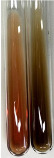	0.36 ± 0.00
**Am Fr**	The appearance of a dark green colour was observed	+	+	−	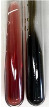	1.13 ± 0.02
**Arv H**	The appearance of a dark green colour and the precipitation of a fine precipitate were observed	+	+	−	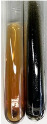	1.00 ± 0.12
**Bv R**	The appearance of a brown colour and the formation of a precipitate were observed	−	−	−	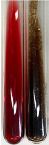	0.53 ± 0.02
**Co F**	A change in colour of the solution to dark green and a jelly-like consistency was observed	+	+	−	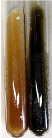	0.75 ± 0.00
**Ea H**	The appearance of a dark green colour was observed	+	+	−	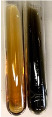	0.42 ± 0.02
**Ep F**	The appearance of a dark green colour was observed	+	+	−	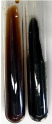	3.17 ± 0.03
**Ep L**	The appearance of a dark green colour was observed	+	+	−	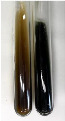	2.20 ± 0.02
**Hp H**	A dark green colour change and precipitation were observed	+	+	−	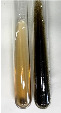	1.47 ± 0.05
**Hr Fr**	The appearance of a dark green colour was observed	+	+	−	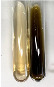	0.52 ± 0.02
**Lc S**	A colour change to dirty yellow was observed	−	−	−	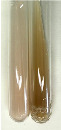	0.13 ± 0.01
**Mc F**	The appearance of a dark green colour and turbidity of the solution were observed	+	+	−	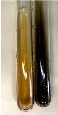	0.50 ± 0.02
**Ob H**	A colour change to dark green was observed and a fine precipitate formed	+	+	−	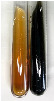	1.44 ± 0.03
**Pm H**	A colour change to dark green was observed	+	+	−	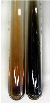	0.92 ± 0.04
**Poa H**	The appearance of a dark green colour was observed	+	+	−	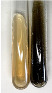	0.36 ± 0.00
**Ps S**	A slight orange colour was observed	−	−	−	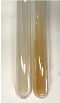	0.07 ± 0.02
**Pta L**	A dark green colour change and turbidity of the solution were observed	+	+	−	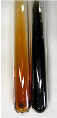	3.11 ± 0.03
**Sg L**	The appearance of a dark green colour and turbidity of the solution were observed	+	+	−	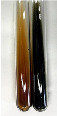	1.65 ± 0.04
**So R**	The appearance of a dark green colour and the formation of a precipitate were observed	+	+	−	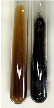	0.88 ± 0.02
**To F**	The appearance of a dark green colour was observed	+	+	−	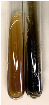	0.55 ± 0.00
**To L**	The appearance of a dark green colour was observed	+	+	−	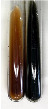	1.14 ± 0.02
**To R**	The appearance of an olive colour was observed	−	+	−	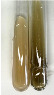	0.18 ± 0.01
**Tp F**	The appearance of a dark green colour was observed	+	+	−	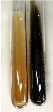	0.82 ± 0.04
**Ur L**	The appearance of a dark green colour was observed	+	+	−	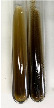	0.13 ± 0.01
**Ur R**	The appearance of a dirty yellow colour was observed	−	−	−	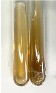	0.09 ± 0.01
**Vo R**	The appearance of a dark green colour and the formation of a precipitate were observed	+	+	−	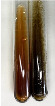	0.46 ± 0.01

+—present; −—not present. Abbreviations: Alv L—aloe leaves; Am Fr—black chokeberry fruits; Arv H—common mugwort herb; Bv R—beetroot roots; Co F—common marigold flowers; Ea H—field horsetail herb; Ep F—purple coneflower flowers; Ep L—purple coneflower leaves; Hp H—St. John’s wort herb; Hr Fr—sea-buckthorn fruits; Lc S—red lentil seeds; Mc F—chamomile flowers; Ob H—basil herb; Pm H—broadleaf plantain herb; Poa H—common knotgrass herb; Ps S—pea seeds; Pta L—common bracken leaves; Sg L—giant goldenrod leaves; So R—comfrey roots; To F—common dandelion flowers; To L—common dandelion leaves; To R—common dandelion roots; Tp F—red clover flowers; Ur L—nettle leaves; Ur R—nettle roots; Vo R—valerian roots.

**Table 2:** The results of lead acetate test, zinc hydrochloride test, and Shinoda test (PC—phenolic compounds, TN—tannins, FD—flavonoids).

Method	Lead Acetate Test					Zinc Hydrochloride Test				Shinoda Test			
Extract	Observation	PC	TN	FD	Photo	Observation	PC	FD	Photo	Observation	PC	FD	Photo
**Alv L**	Precipitation of a white precipitate and a change in colour of the solution to beige-milky were observed	+	+	−	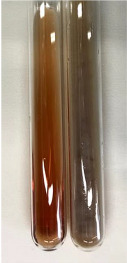	Change of colour of the solution to light green, foaming	−	−	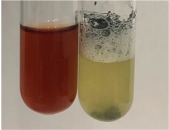	The colour of the solution changes to orange, the formation of foam in the upper part of the solution	+	−	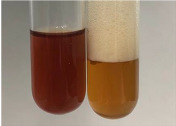
**Am Fr**	A colour change to bottle green was observed	−	−	−	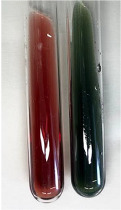	The colour of the solution changes to pink	−	+	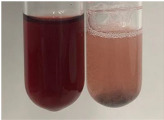	The colour of the solution changes to bright red	−	+	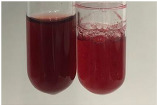
**Arv H**	Precipitation and a colour change to olive green were observed	−/+	+	+	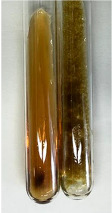	Change of the colour of the solution to light yellow-green, formation of precipitate and foam	−/+	−	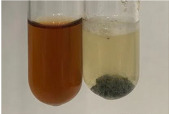	Change of colour of the solution to light orange, foam formation	+	−	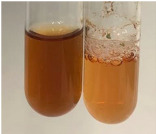
**Bv R**	A change in colour of the solution to strawberry colour and precipitation were observed	+	+	−	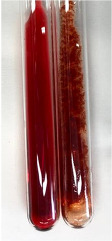	The colour of the solution changes to yellow, the release of foam	+	−	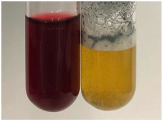	The colour of the solution changes to dark red	−	+	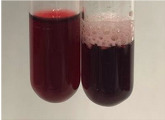
**Co F**	Precipitation of a jelly-like precipitate and colour change to dirty yellow were observed	−/+	+	+	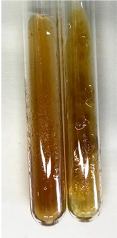	The colour of the solution changes to yellow, the formation of a grey precipitate in the upper part of the tube	+	−	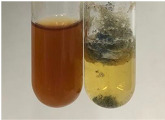	The formation of a orange jelly-like consistency	−/+	−	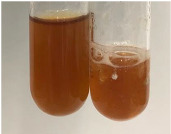
**Ea H**	A colour change to dirty yellow and precipitation were observed	−/+	+	+	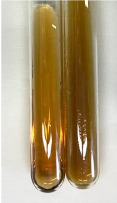	Change of the colour of the solution to lemon, release of foam	−/+	−	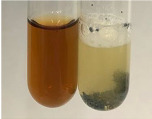	The colour of the solution changes to bright orange	+	−	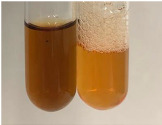
**Ep F**	Turbidity of the solution and an olive colour were observed	−	−	−	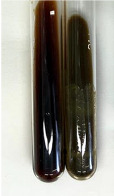	The colour of the solution changes to orange	+	−	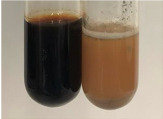	The colour of the solution changes to red	−	+	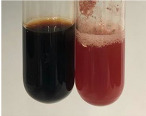
**Ep L**	A light green colour was observed and a slight turbidity appeared	−	−	−	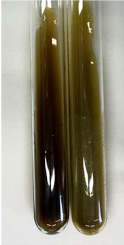	The colour of the solution changes to yellow-orange	+	−	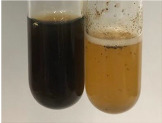	The colour of the solution changes to orange	+	−	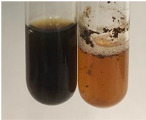
**Hp H**	A colour change to olive green and turbidity of the solution were observed	−	−	−	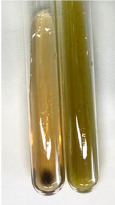	Discolouration of the solution, formation of foam and precipitate	−	−	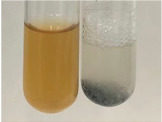	Change of colour of the solution to light orange, foam formation	−/+	−	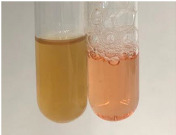
**Hr Fr**	The solution turned yellow	−	−	−	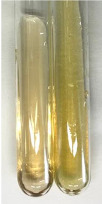	Change of the colour of the solution to cloudy grey, release of foam	−	−	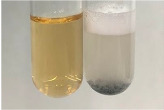	The colour of the solution changes to pink-red	−	+	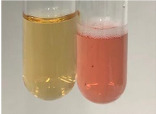
**Lc S**	A colour change of one degree (darker) was observed	−	−	−	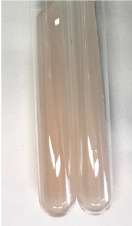	The colour of the solution changes to pale yellow, the formation of foam	−/+	−	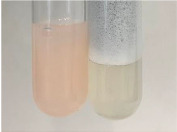	The colour of the solution changes to light pink	−	+	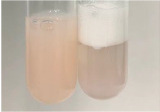
**Mc F**	Precipitation of a white precipitate was observed	+	+	−	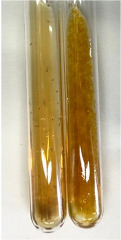	Change of the colour of the solution to light lemon, precipitation of a delicate precipitate	−/+	−	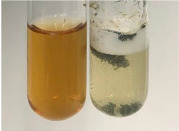	no colour change, evolution of foam in the upper part of the solution	−/+	−	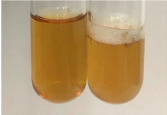
**Ob H**	Precipitation of a white precipitate and a colour change to olive green were observed	+	+	−	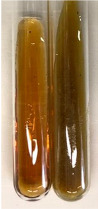	The colour of the solution changes to yellow-green, the release of foam	−/+	−	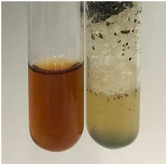	The colour of the solution changes to bright orange	+	−	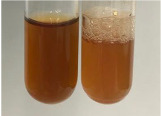
**Pm H**	Precipitation of a white precipitate and a colour change to olive green were observed	+	+	−	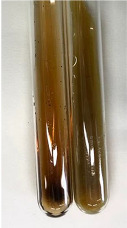	Change of colour of the solution to yellow-green, foaming	−/+	−	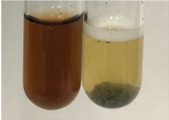	The colour of the solution changes to orange	+	−	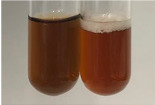
**Poa H**	A colour change to intense yellow and precipitation were observed	−	+	+	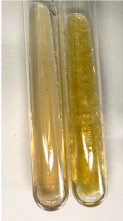	Precipitation of zinc (grey), discolouration of the solution	−	−	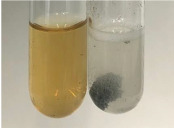	The colour of the solution changes to bright orange	+	−	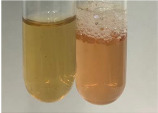
**Ps S**	No changes were observed	−	−	−	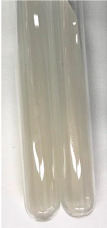	Exudation of a large amount of foam and its deposition on the walls of the test tube	−	−	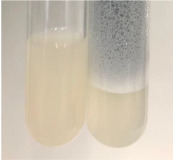	The colour of the solution changes to light pink	−	+	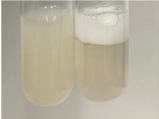
**Pta L**	Precipitation was observed	−	+	+	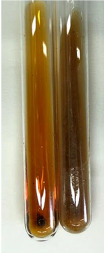	The colour of the solution changes to light yellow	−/+	−	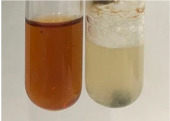	The colour of the solution changes to yellow, the release of foam	+	−	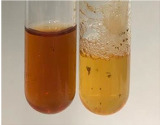
**Sg L**	A colour change to olive green was observed	−	−	−	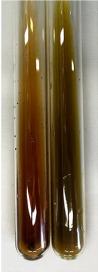	The colour of the solution changes to yellow, the release of foam	+	−	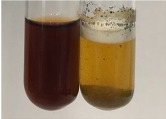	The colour of the solution changes to amber-orange	+	−	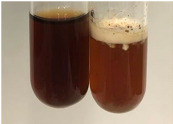
**So R**	The appearance of a gelatinous form and a change in the colour of the solution to brown were observed	−	−	−	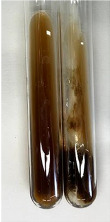	Formation of a gelatinous consistency	−	−	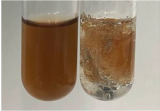	Formation of a gelatinous consistency	−	−	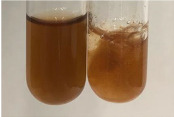
**To F**	A colour change to dirty yellow and precipitation were observed	−	+	+	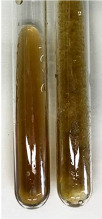	The colour of the solution changes to pale yellow, the formation of foam	−/+	−	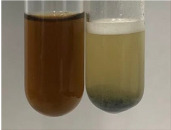	The colour of the solution changes to bright orange	+	−	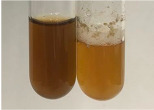
**To L**	A colour change to olive and precipitation were observed	−/+	+	+	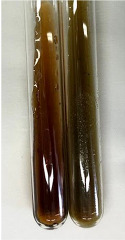	The colour of the solution changes to pale yellow, the formation of foam	−/+	−	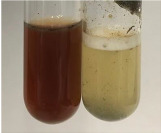	The colour of the solution changes to bright orange	+	−	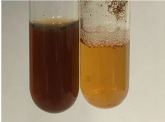
**To R**	A change in the colour of the solution to yellow and the formation of a fine precipitate were observed	−/+	+	+	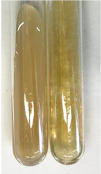	Turbidity of the solution	−	−	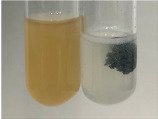	The colour of the solution changes to light yellow	−/+	−	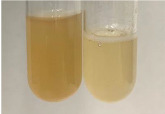
**Tp F**	A colour change of the solution to olive green was observed	−	−	−	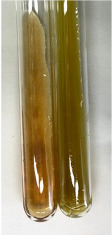	Change of colour of the solution to light orange, precipitation of a fine precipitate	+	−	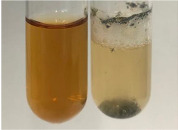	The colour of the solution changes to orange-red	−	+	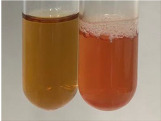
**Ur L**	A change in colour to lemon was observed	−	−	−	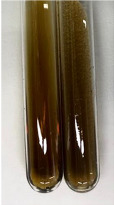	Change of colour of the solution to yellow- orange, foaming	+	−	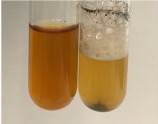	The colour of the solution changes to brown-orange	−/+	−	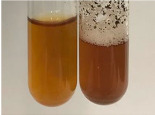
**Ur R**	The appearance of a white precipitate was observed	+	+	−	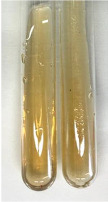	Change of the colour of the solution to lemon, release of foam	+	−	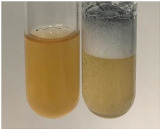	The colour of the solution changes to light yellow	−/+	−	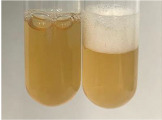
**Vo R**	Precipitation was observed	+	+	+	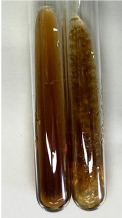	Change of colour of the solution to yellow, separation of a grey precipitate	+	−	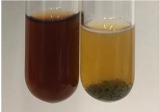	The colour of the solution changes to amber-orange	+	−	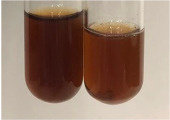

+—present; −—not present; −/+—not obvious. Abbreviations: Alv L—aloe leaves; Am Fr—black chokeberry fruits; Arv H—common mugwort herb; Bv R—beetroot roots; Co F—common marigold flowers; Ea H—field horsetail herb; Ep F—purple coneflower flowers; Ep L—purple coneflower leaves; Hp H—St. John’s wort herb; Hr Fr—sea-buckthorn fruits; Lc S—red lentil seeds; Mc F—chamomile flowers; Ob H—basil herb; Pm H—broadleaf plantain herb; Poa H—common knotgrass herb; Ps S—pea seeds; Pta L—common bracken leaves; Sg L—giant goldenrod leaves; So R—comfrey roots; To F—common dandelion flowers; To L—common dandelion leaves; To R—common dandelion roots; Tp F—red clover flowers; Ur L—nettle leaves; Ur R—nettle roots; Vo R—valerian roots.

**Table 3:** The results of gelatin test, alkaline reagent test, and bromine water test (TN—tannins, FD—flavonoids, GS—glycosides, SG—sugars).

Method	Gelatin Test			Alkaline Reagent Test				Bromine Water Test				
Extract	Observation	TN	Photo	Observation	TN	FD	Photo	Observation	TN	GS	SG	Photo
**Alv L**	The formation of two phases: dark and light orange	−	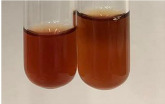	Brown-orange colour of the solution and a yellow glow. After addition of HCl: Orange colour of the solution and formation of a dark orange glow	−	−	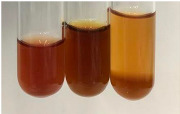	The colour of the solution changes to amber-orange	−	−	−	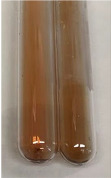
**Am Fr**	The formation of two phases: dark brown and pink with a delicate precipitate	−	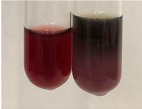	No yellow glow/green colour of the solution. After addition of HCl: The formation of 3 phases: brown, red and black	−	−	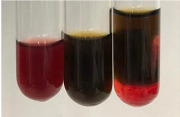	The colour of the solution changes to orange	−	−	−	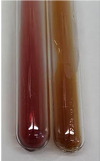
**Arv H**	The colour of the solution changes to olive green	−	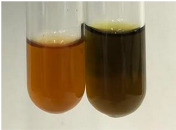	Brown-orange colour of the solution and a yellow glow. After addition of HCl: Orange colour of the solution	−	−	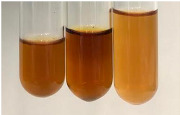	Change of colour of the solution to orange, precipitation	+	−/+	−	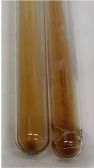
**Bv R**	The formation of two phases: raspberry and dark-burgundy	−	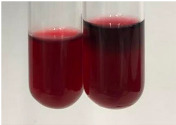	Orange colour of the solution. After addition of HCl: Red-orange colour of the solution	−	−	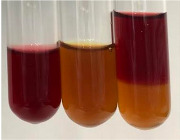	The colour of the solution changes to orange	−	−	−	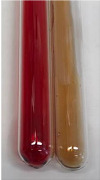
**Co F**	The colour of the solution changes to an intense orange	−	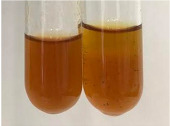	Orange colour of the solution and a yellow glow. After addition of HCl: Orange-amber colour of the solution and formation of a yellow glow	−	−	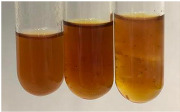	No changes were observed	−	−	−	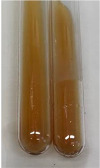
**Ea H**	The colour of the solution changes to an intense orange	−	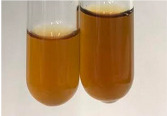	Orange colour of the solution and a yellow glow. After addition of HCl: Yellow solution and orange precipitate	+	−	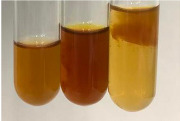	The colour of the solution changes to dark orange	−	−	−	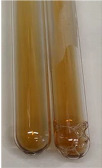
**Ep F**	The colour of the solution changes to brown, the formation of a delicate precipitate	−	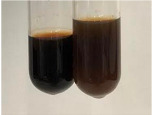	The appearance of a yellow glow. After addition of HCl: The formation of 3 phases: brick red, orange, black	−	−	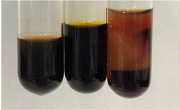	No changes were observed	−	−	−	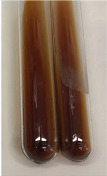
**Ep L**	The formation of three phases: dark brown, brown and dark burgundy with a delicate precipitate	−	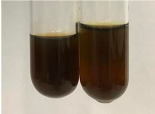	The appearance of a yellow glow. After addition of HCl: Orange colour of the solution and slight dispersion of the phases	−	−	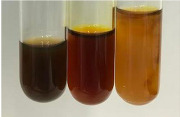	The colour of the solution changes to amber-orange	−	−	−	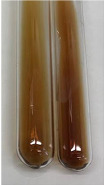
**Hp H**	The colour of the solution changes to orange, the formation of a delicate white precipitate	+	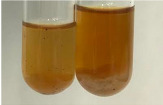	Brown colour of the solution and a yellow glow. After addition of HCl: Orange- yellow-brown colour of the solution and formation of a yellow glow	−	−	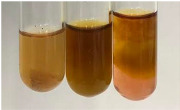	The colour of the solution changes to orange	−	−	−	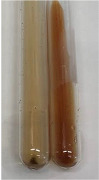
**Hr Fr**	The colour of the solution changes to cloudy yellow	−	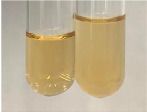	The appearance of a yellow colour. After addition of HCl: The formation of 2 phases: light yellow and intense yellow	−	−	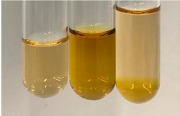	The colour of the solution changes to dark yellow	−	−	−	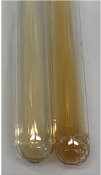
**Lc S**	Precipitation of a powdery pink precipitate	+	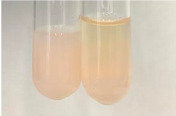	Lemon colour of the solution. After addition of HCl: Lemon coloured solution and light pink precipitate	+	−	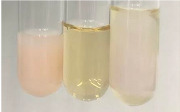	Change of colour of the solution to lemon	−	−	−	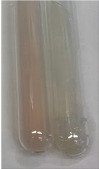
**Mc F**	The formation of two phases: dark and light orange	−	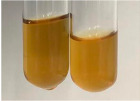	Orange colour of the solution and yellow glow. After addition of HCl: Orange- yellow colour of the solution and formation of a yellow glow	−	−	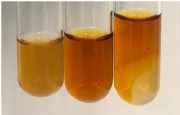	A change in the colour tone of the solution was observed	−	−	−	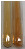
**Ob H**	The colour of the solution changes to brown	−	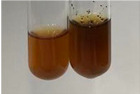	Brown-orange colour of the solution and a yellow glow. After addition of HCl: Orange-brown colour of the solution and formation of a yellow glow	−	−	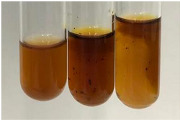	The colour of the solution changes to orange	−	−	−	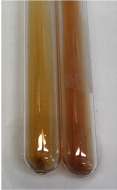
**Pm H**	Precipitation of a dark maroon solid	−	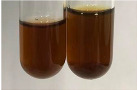	Amber colour of the solution and a yellow glow. After addition of HCl: Orange-brown colour of the solution	−	−	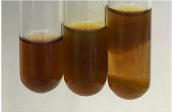	The colour of the solution changes to orange	−	−	−	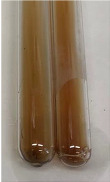
**Poa H**	No changes were observed	−	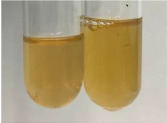	Orange colour of the solution and yellow glow. After addition of HCl: Yellow- orange colour of the solution and finely dispersed precipitate	−	−	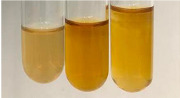	The colour of the solution changes to an intense yellow	−	−	−	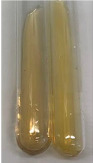
**Ps S**	Formation of a fine white precipitate	+	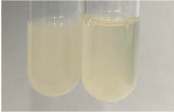	Lemon colour of the solution. After addition of HCl: Lemon coloured solution and white precipitate	−	−	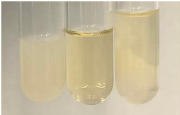	Precipitation of a fine precipitate	+	−	−	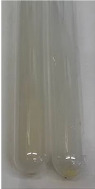
**Pta L**	The colour of the solution changes to red-orange	−	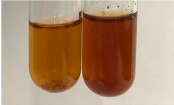	Brown-red colour of the solution. After addition of HCl: Yellow-brown colour of the solution and formation of a fine precipitate	+	−	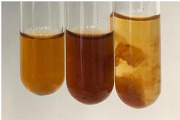	The colour of the solution changes to orange	−	−	−	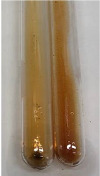
**Sg L**	The formation of two phases: brown and orange	−	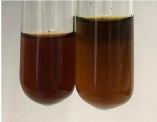	Brown-red colour of the solution and a yellow glow. After addition of HCl: Orange-brown colour of the solution	−	−	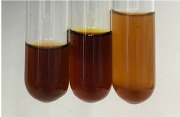	The colour of the solution changes to orange	−	−	−	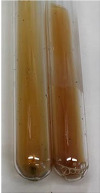
**So R**	No changes were observed	−	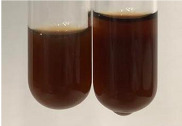	Brown-red colour of the solution and a yellow glow. After addition of HCl: Amber-orange colour of the solution	−	−	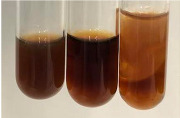	The appearance of a jelly-like consistency	−	−	−	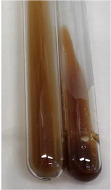
**To F**	No changes were observed	−	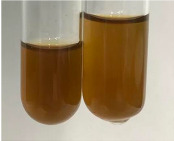	Orange colour of the solution and yellow glow. After addition of HCl: Orange colour of the solution and formation of a dark precipitate	+	−	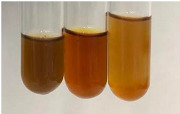	No changes were observed	−	−	−	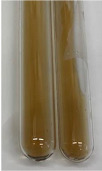
**To L**	The formation of two phases: brown and orange	−	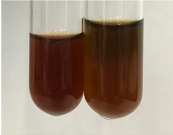	Red-orange colouration and yellow glow. After addition of HCl: Orange-brown colour of the solution	−	−	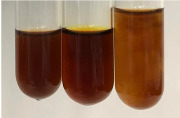	The appearance of an orange glow	−	−	−	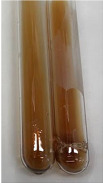
**To R**	Formation of two phases: cloudy yellow and yellow-orange	−	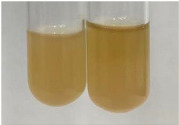	Yellow colour of the solution. After addition of HCl: Lemon yellow colour of the solution	−	−	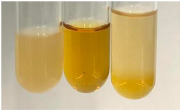	Change of colour of the solution to lemon	−	−	−	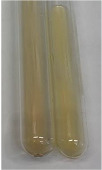
**Tp F**	The formation of two phases: dark and light orange	−	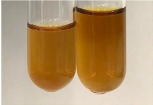	Orange colour of the solution and yellow glow. After addition of HCl: Orange colour of the solution	−	−	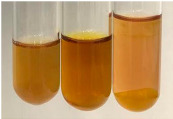	The colour of the solution changes to orange	−	−	−	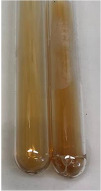
**Ur L**	No changes were observed	−	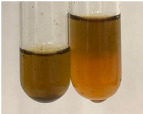	Orange colour. After addition of HCl: Slight orange colour and dispersed precipitate formation	+	−	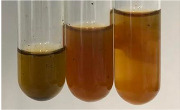	The colour of the solution changes to yellow	−	−	−	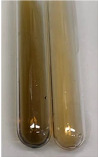
**Ur R**	No changes were observed	−	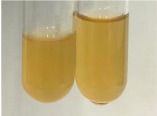	Intense yellow colour. After addition of HCl: The formation of 2 phases: lemon and intense yellow	−	−	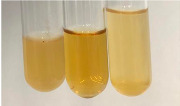	Change of colour of the solution to lemon, precipitation of a precipitate	+	+	−	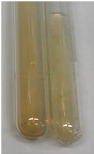
**Vo R**	No changes were observed	−	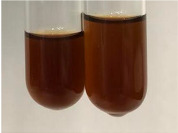	Brown-red colour of the solution and a yellow glow. After addition of HCl: Amber-orange colour of the solution	−	−	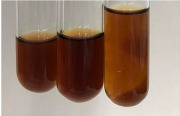	The colour of the solution changes to orange-yellow	−	−	−	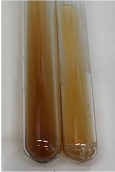

+—present; −—not present; −/+—not obvious. Abbreviations: Alv L—aloe leaves; Am Fr—black chokeberry fruits; Arv H—common mugwort herb; Bv R—beetroot roots; Co F—common marigold flowers; Ea H—field horsetail herb; Ep F—purple coneflower flowers; Ep L—purple coneflower leaves; Hp H—St. John’s wort herb; Hr Fr—sea-buckthorn fruits; Lc S—red lentil seeds; Mc F—chamomile flowers; Ob H—basil herb; Pm H—broadleaf plantain herb; Poa H—common knotgrass herb; Ps S—pea seeds; Pta L—common bracken leaves; Sg L—giant goldenrod leaves; So R—comfrey roots; To F—common dandelion flowers; To L—common dandelion leaves; To R—common dandelion roots; Tp F—red clover flowers; Ur L—nettle leaves; Ur R—nettle roots; Vo R—valerian roots.

**Table 4:** The results of potassium dichromate test and HCl test (TN—tannins).

Method	Potassium Dichromate Test			HCl Test (Phlobatannins)		
Extract	Observation	TN	Photo	Observation	TN	Photo
**Alv L**	The colour of the solution changes to red	−	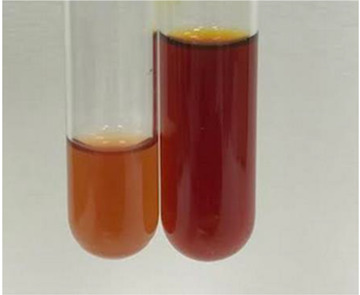	A colour change of the solution to yellow was observed	−	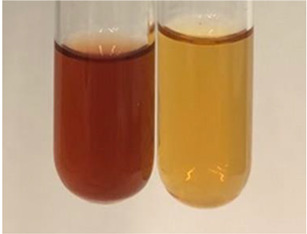
**Am Fr**	The colour of the solution changes to a dark colour	−	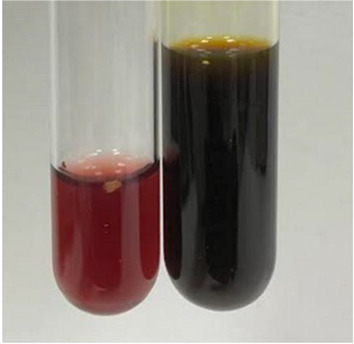	A change in colour tone to a brighter red was observed	−	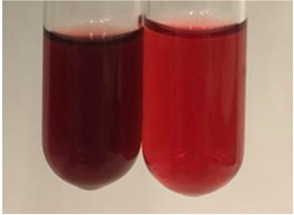
**Arv H**	The colour of the solution changes to a dark colour	−	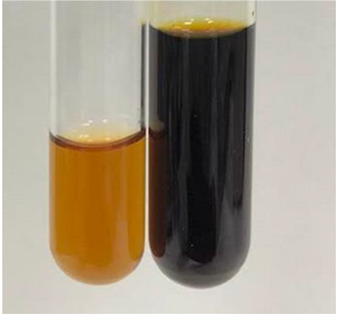	A colour change of the solution to light orange was observed	−	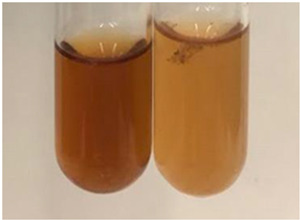
**Bv R**	The colour of the solution changes to dark maroon	−	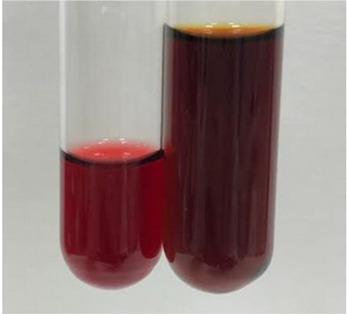	A change in the colour of the solution to orange was observed, the separation of a fine precipitate	−/+	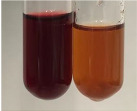
**Co F**	The appearance of 2 phases was observed: orange-brown and red	−	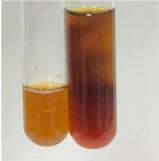	A change in the colour of the solution to orange-yellow was observed, precipitation of a precipitate in the upper part of the tube	−/+	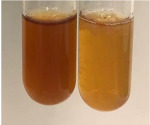
**Ea H**	The colour of the solution changes to brown-red	−	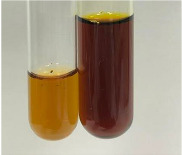	A colour change of the solution to yellow was observed	−	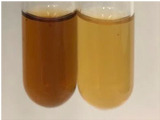
**Ep F**	The colour of the solution changes to a dark colour	−	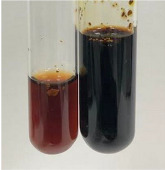	A colour change of the solution to orange was observed	−	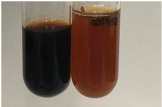
**Ep L**	The colour of the solution changes to a dark colour	−	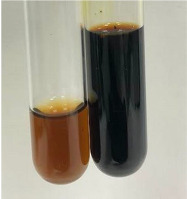	A colour change of the solution to yellow-orange was observed	−	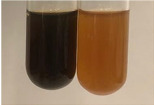
**Hp H**	The colour of the solution changes to a dark colour	−	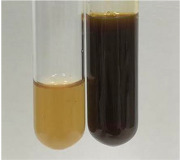	A colour change of the solution to light orange was observed	−	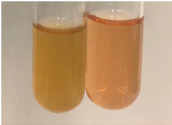
**Hr Fr**	The colour of the solution changes to orange-brick	−	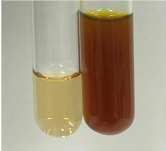	A change in colour tone to a brighter yellow was observed	−	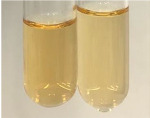
**Lc S**	The colour of the solution changes to intense orange	−	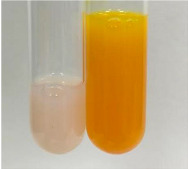	Discolouration of the solution was observed	−	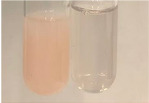
**Mc F**	The colour of the solution changes to orange	−	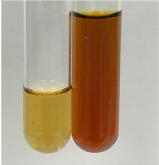	A change in the colour tone of the solution to a bright yellow was observed	−	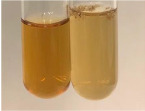
**Ob H**	The colour of the solution changes to a dark colour	−	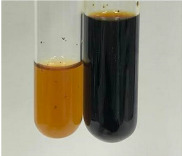	A colour change of the solution to orange was observed	−	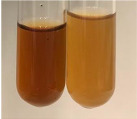
**Pm H**	The colour of the solution changes to a dark colour	−	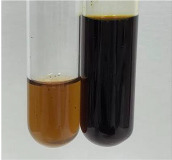	A colour change of the solution to olive green was observed	−	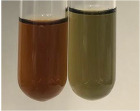
**Poa H**	The colour of the solution changes to brown-orange	−	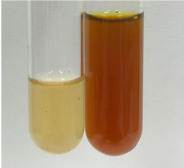	A change in the colour tone of the solution to bright yellow was observed	−	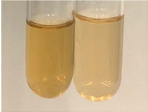
**Ps S**	The colour of the solution changes to intense orange	−	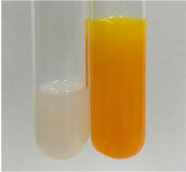	Discolouration of the solution was observed	−	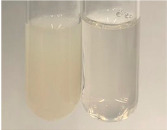
**Pta L**	The colour of the solution changes to a brown-orange colour	−	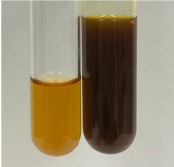	A colour change of the solution to light orange was observed	−	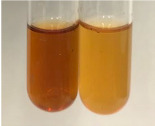
**Sg L**	The colour of the solution changes to a dark colour	−	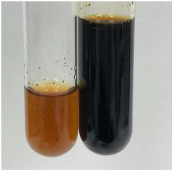	A colour change of the solution to orange was observed	−	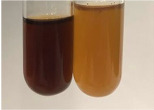
**So R**	The colour of the solution changes to a dark colour	−	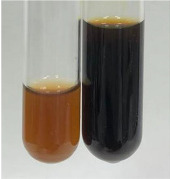	A change in the colour of the solution to light yellow was observed, the separation of a delicate precipitate	+	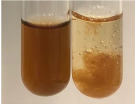
**To F**	The colour of the solution changes to dark brown	−	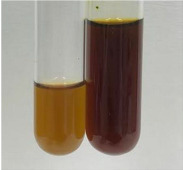	A colour change of the solution to orange-yellow was observed	−	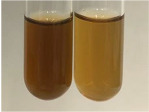
**To L**	The colour of the solution changes to a dark colour	−	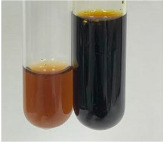	A colour change of the solution to orange-yellow was observed	−	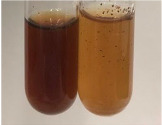
**To R**	The colour of the solution changes to orange	−	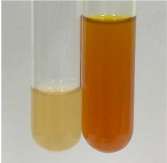	A change in the colour tone of the solution to bright yellow was observed	−	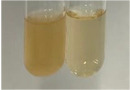
**Tp F**	The colour of the solution changes to red-brown	−	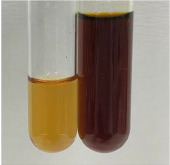	A colour change of the solution to light orange was observed	−	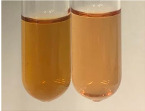
**Ur L**	The colour of the solution changes to orange-brick red	−	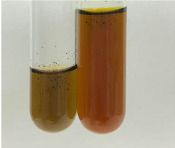	A change in colour tone to a brighter yellow was observed	−	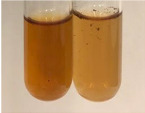
**Ur R**	The colour of the solution changes to intense orange	−	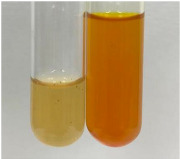	A change in the colour of the solution to a clear lemon colour was observed	−	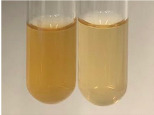
**Vo R**	The colour of the solution changes to red-orange	−	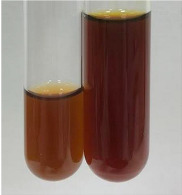	Appearance of a maroon precipitate in the upper part of the tube and a change in the colour of the solution to orange	+	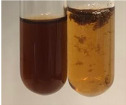

+—present; −—not present; −/+—not obvious. Abbreviations: Alv L—aloe leaves; Am Fr—black chokeberry fruits; Arv H—common mugwort herb; Bv R—beetroot roots; Co F—common marigold flowers; Ea H—field horsetail herb; Ep F—purple coneflower flowers; Ep L—purple coneflower leaves; Hp H—St. John’s wort herb; Hr Fr—sea-buckthorn fruits; Lc S—red lentil seeds; Mc F—chamomile flowers; Ob H—basil herb; Pm H—broadleaf plantain herb; Poa H—common knotgrass herb; Ps S—pea seeds; Pta L—common bracken leaves; Sg L—giant goldenrod leaves; So R—comfrey roots; To F—common dandelion flowers; To L—common dandelion leaves; To R—common dandelion roots; Tp F—red clover flowers; Ur L—nettle leaves; Ur R—nettle roots; Vo R—valerian roots.

**Table 5:** The results of NaOH test and H_2_SO_4_ test (AC—anthocyanins, CM—coumarins, FL—flavones).

Method	NaOH Test					H_2_SO_4_ Test			
Extract	Observation	AC	CM	FL	Photo	Observation	AC	FL	Photo
**Alv L**	The colour of the solution changes to orange	−	−	−	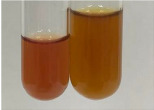	The colour of the solution changes to orange	+	+	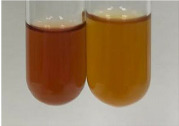
**Am Fr**	Appearance of a fine precipitate, colour change of the precipitate to yellow-brown	−	−	−	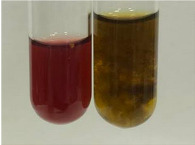	The colour of the solution changes to a more intense red	+	+	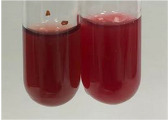
**Arv H**	The colour of the solution changes to orange	−	−	−	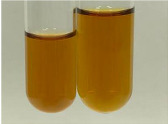	The colour of the solution changes to a cloudy brown-orange	−/+	−/+	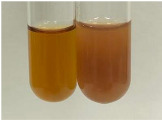
**Bv R**	The colour of the solution changes to orange	−	−	−	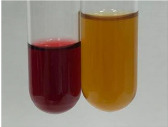	The colour of the solution changes to dark maroon	−	−	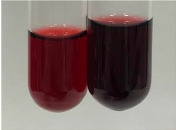
**Co F**	The colour of the solution changes to orange	−	−	−	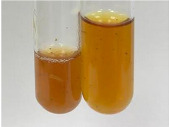	Change of colour of the solution to orange and precipitation of a fine precipitate	−/+	−/+	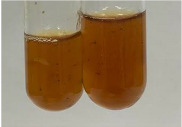
**Ea H**	The colour of the solution changes to orange-yellow	−	−/+	−/+	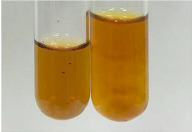	Slight turbidity of the solution, orange colour of solution	−/+	−/+	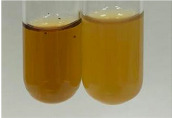
**Ep F**	The appearance of a yellow glow	−	−	−	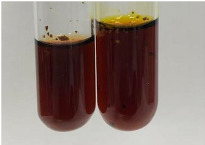	Turbidity of the solution, cloudy red-brown colour of solution	−/+	−/+	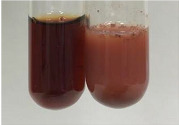
**Ep L**	The colour of the solution changes to orange-yellow	−	−/+	−/+	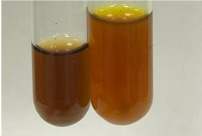	Turbidity of the solution, brown-orange colour of solution	−/+	−/+	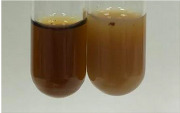
**Hp H**	The colour of the solution changes to yellow-brown	−	−/+	−/+	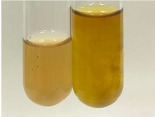	The colour of the solution changes to cloudy red	+	+	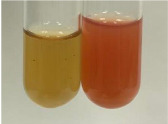
**Hr Fr**	The colour of the solution changes to an intense yellow	−	+	+	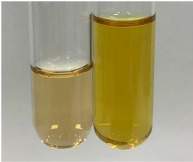	No changes were observed	−	−	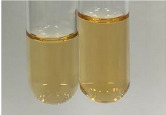
**Lc S**	The colour of the solution changes to light lemon	−	−/+	−/+	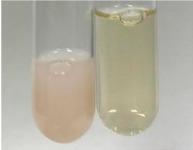	Two phases are created: pink and light lemon	−	−	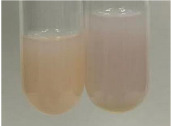
**Mc F**	The colour of the solution changes to yellow	−	+	+	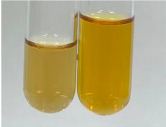	No changes were observed	−	−	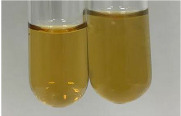
**Ob H**	The colour of the solution changes to orange	−	−	−	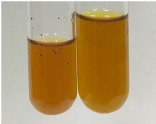	Turbidity of the solution, brown-orange colour of solution	−/+	−/+	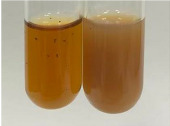
**Pm H**	The colour of the solution changes to orange-yellow	−	−/+	−/+	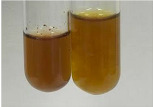	No changes were observed, brown-orange colour of solution	−/+	−/+	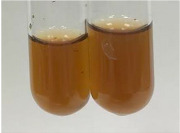
**Poa H**	The colour of the solution changes to yellow	−	+	+	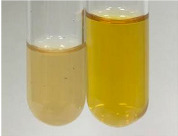	The colour of the solution changes to bright orange	+	+	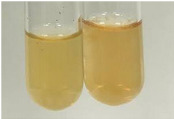
**Ps S**	The colour of the solution changes to light lemon	−	−/+	−/+	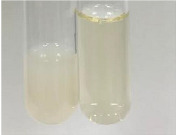	The formation of 2 phases: cloudy and light lemon	−	−	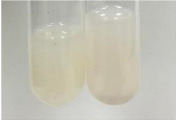
**Pta L**	Orange colour of the solution and the appearance of a fine precipitate	−	−	−	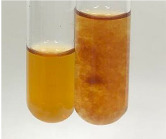	The colour of the solution changes to orange-yellow	+	+	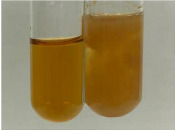
**Sg L**	The colour of the solution changes to orange	−	−	−	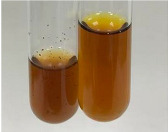	Colour change of the solution to orange-brown	−/+	−/+	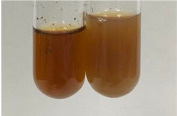
**So R**	The colour of the solution changes to orange	−	−	−	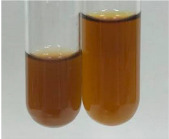	The formation of a jelly-like consistency, orange-brown colour of solution	−/+	−/+	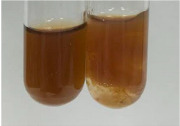
**To F**	The colour of the solution changes to orange	−	−	−	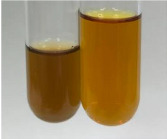	The colour of the solution changes to brown-orange	−/+	−/+	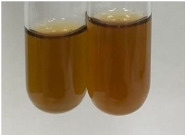
**To L**	The colour of the solution changes to orange	−	−	−	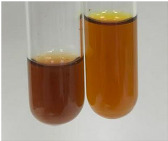	Precipitation formation, brown-orange colour of solution	−	−	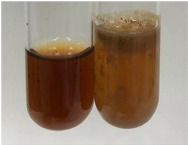
**To R**	The colour of the solution changes to yellow	−	+	+	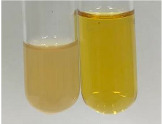	No changes were observed	−	−	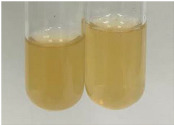
**Tp F**	The colour of the solution changes to orange-yellow	−	−/+	−/+	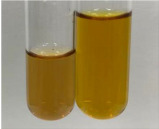	The colour of the solution changes to cloudy orange	+	+	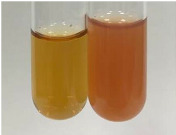
**Ur L**	Change of colour of the solution to orange-yellow, precipitation of a fine precipitate	−	−/+	−/+	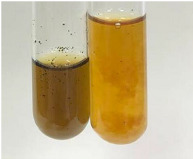	The colour of the solution changes to orange	+	+	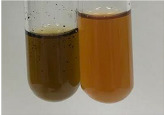
**Ur R**	The colour of the solution changes to lemon	−	−/+	−/+	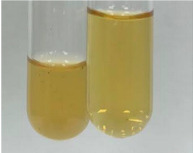	No changes were observed	−	−	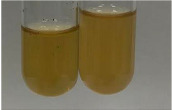
**Vo R**	The colour of the solution changes to orange	−	−	−	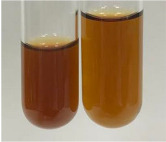	No changes were observed	−	−	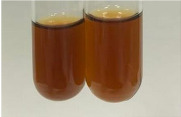

+—present; −—not present; −/+—not obvious. Abbreviations: Alv L—aloe leaves; Am Fr—black chokeberry fruits; Arv H—common mugwort herb; Bv R—beetroot roots; Co F—common marigold flowers; Ea H—field horsetail herb; Ep F—purple coneflower flowers; Ep L—purple coneflower leaves; Hp H—St. John’s wort herb; Hr Fr—sea-buckthorn fruits; Lc S—red lentil seeds; Mc F—chamomile flowers; Ob H—basil herb; Pm H—broadleaf plantain herb; Poa H—common knotgrass herb; Ps S—pea seeds; Pta L—common bracken leaves; Sg L—giant goldenrod leaves; So R—comfrey roots; To F—common dandelion flowers; To L—common dandelion leaves; To R—common dandelion roots; Tp F—red clover flowers; Ur L—nettle leaves; Ur R—nettle roots; Vo R—valerian roots.

**Table 6:** The results of aluminium chloride test, ammonium test, ammonia and H_2_SO_4_ test, and DNPH tests (FD—flavonoids, VC—Vitamin C).

Method	Aluminium Chloride Test			Ammonium Test			Ammonia and H_2_SO_4_ Test			DNPH Test		
Extract	Observation	FD	Photo	Observation	FD	Photo	Observation	FD	Photo	Observation	VC	Photo
**Alv L**	Red-orange colour of the solution; Orange colour of the solution and precipitation of a fine precipitate	−/+	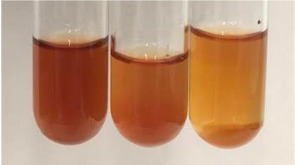	The colour of the solution changes to orange with a yellow glow	−/+	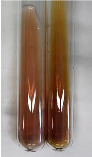	The colour of the solution changes to orange	−	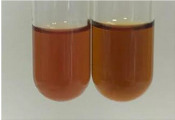	The colour of the solution changes to brown-orange	−	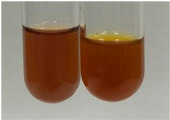
**Am Fr**	Purple-raspberry colour; Formation of 2 phases: red and green	−	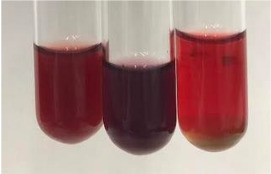	The colour of the solution changes to olive green	−	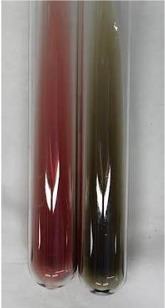	The colour of the solution changes to orange-amber	−	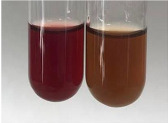	Turbidity of the solution, change to a lighter colour	−	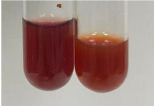
**Arv H**	Green-brown colour of the solution; Orange-yellow colour of the solution	−/+	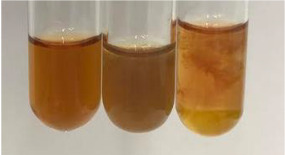	The colour of the solution changes to olive green	−	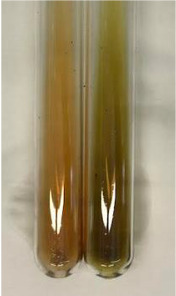	The colour of the solution changes to light green	−	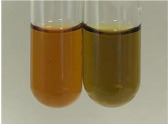	The colour of the solution changes to an intense orange	−	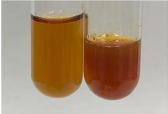
**Bv R**	Red colour of the solution; Formation of 2 phases: red and orange	−/+	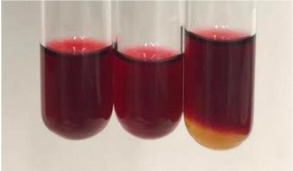	The colour of the solution changes to maroon	−	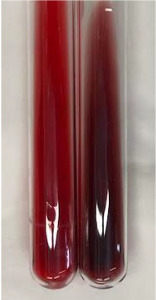	The colour of the solution changes to orange	−	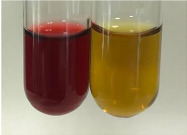	The colour of the solution changes to maroon	−	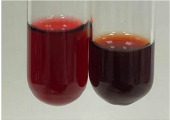
**Co F**	Orange-yellow colour of the solution; Orange-yellow colour of the solution	−/+	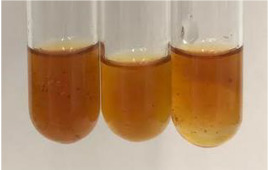	The colour of the solution changes to orange-yellow	−/+	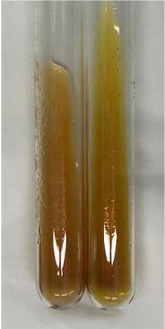	The colour of the solution changes to yellow	+	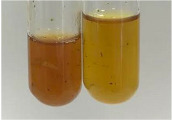	The colour of the solution changes to an intense red-orange	−	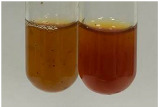
**Ea H**	Orange colour of the solution; Yellow colour of the solution and precipitation of an orange precipitate	−/+	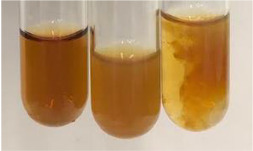	The colour of the solution changes to brown with a yellow glow	−/+	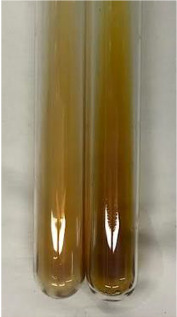	The colour of the solution changes to yellow	+	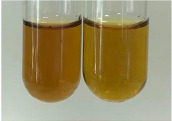	The colour of the solution changes to an intense orange	−	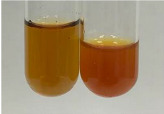
**Ep F**	Formation of 2 phases: yellow-brown and brown; Formation of 2 phases: yellow-brown and orange with a precipitate	−/+	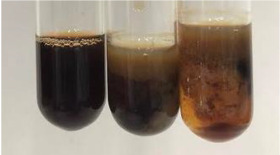	The colour of the solution changes to dark brown with a yellow glow	−/+	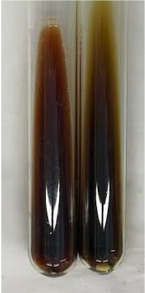	The colour of the solution changes to dark brown	−	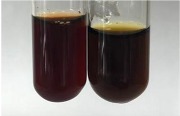	Appearance of a fine white precipitate	−/+	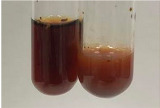
**Ep L**	Olive green colour of the solution; Orange-brown colour of the solution	−/+	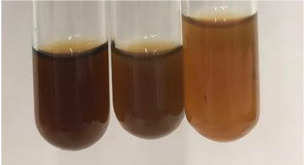	The colour of the solution changes to brown with a yellow glow	−/+	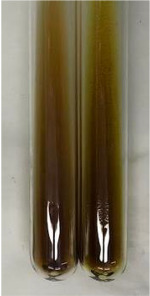	The colour of the solution changes to olive green	−	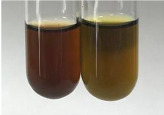	Turbidity of the solution	−	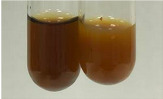
**Hp H**	Yellow-green colour of the solution; Orange-yellow colour of the solution and precipitation of a fine precipitate	−/+	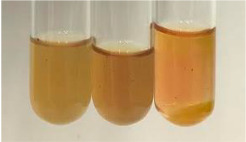	The colour of the solution changes to orange-yellow	−/+	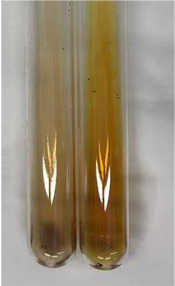	The colour of the solution changes to orange	−	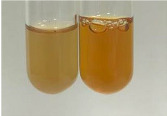	The colour of the solution changes to an intense orange	−	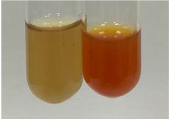
**Hr Fr**	Yellow colour of the solution; Yellow colour of the solution;	−/+	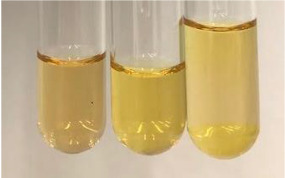	The colour of the solution changes to an intense yellow	+	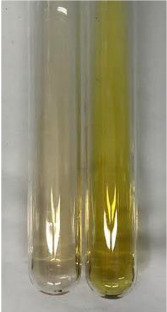	The colour of the solution changes to yellow	+	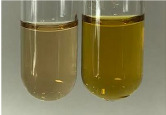	The colour of the solution changes to an intense orange	−	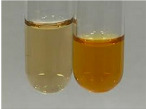
**Lc S**	Salmon-coloured cloudy solution; Lemon colour of the solution and precipitation of a delicate pink precipitate	+	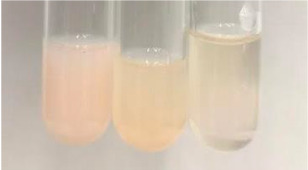	The colour of the solution changes to lemon	−	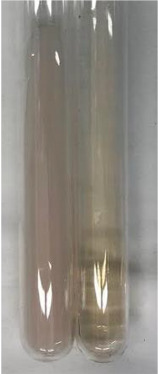	The colour of the solution changes to lemon	−/+	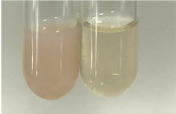	The colour of the solution changes to yellow, the appearance of a white precipitate	+	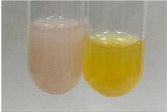
**Mc F**	Yellow colour of the solution; Red-yellow/pale yellow solution	−/+	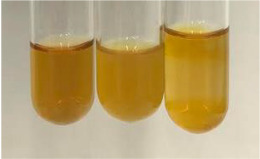	The colour of the solution changes to orange-yellow	−/+	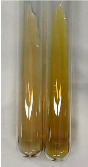	The colour of the solution changes to yellow	+	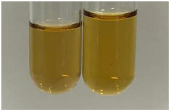	The colour of the solution changes to orange	−	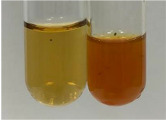
**Ob H**	Orange-brown colour of the solution; Orange-yellow-brown colour of the solution and precipitation of a fine precipitate	−/+	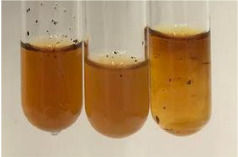	The colour of the solution changes to brown with a yellow glow	−/+	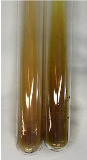	The colour of the solution changes to yellow-brown	−/+	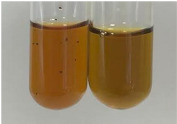	Turbidity of the solution	−	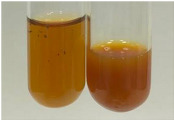
**Pm H**	Orange-brown colour of the solution; Orange-brown colour of the solution	−/+	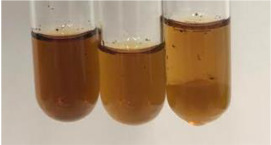	The colour of the solution changes to brown with a yellow glow	−/+	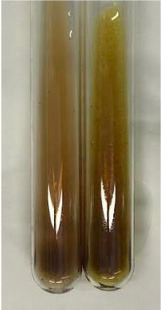	The colour of the solution changes to yellow-brown	−/+	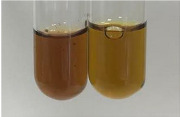	Changing the colour of the solution to a darker shade	−	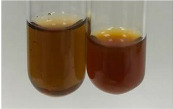
**Poa H**	Yellow colour of the solution; Yellow colour of the solution and precipitation of a fine precipitate	−/+	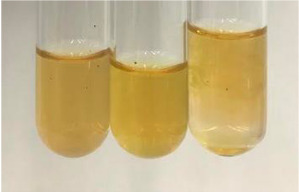	The colour of the solution changes to an intense yellow	+	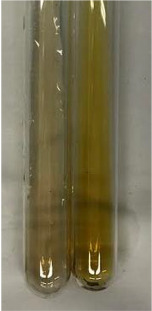	The colour of the solution changes to yellow	−/+	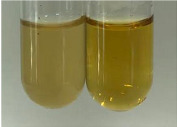	The colour of the solution changes to orange		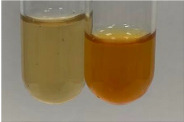
**Ps S**	Cloudy lemon colour of the solution; Cloudy lemon colour of the solution and precipitation	+	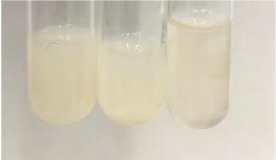	The colour of the solution changes to lemon	−	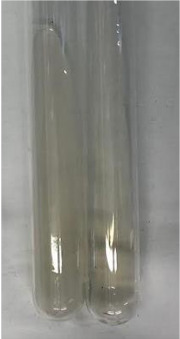	The solution became clear, no colour change	−	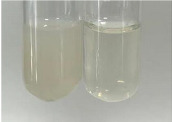	The colour of the solution changes to yellow, the appearance of a white precipitate	+	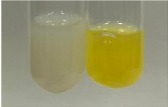
**Pta L**	Orange colour of the solution; Yellow-orange colour of the solution and precipitation of an orange precipitate	−/+	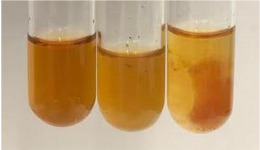	The colour of the solution changes to orange	−	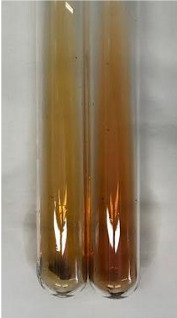	The colour of the solution changes to orange	−	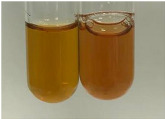	The colour of the solution changes to orange	−	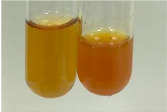
**Sg L**	Olive green solution and precipitation; Formation of 2 phases: olive green and orange- yellow, precipitation	−/+	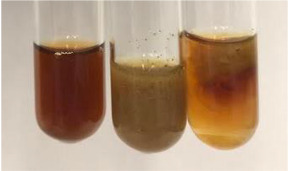	The colour of the solution changes to brown with a yellow glow	−/+	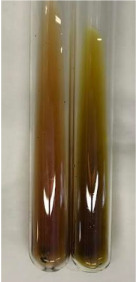	The colour of the solution changes to yellow-brown	−/+	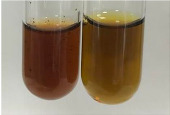	Turbidity of the solution	−	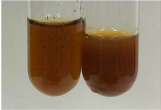
**So R**	Gelatinous brown consistency of the solution; Formation of 2 phases: brown and yellow, the solution took on a jelly-like consistency	−/+	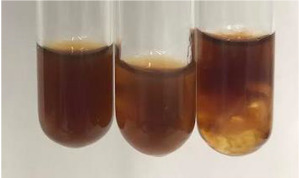	The colour of the solution changes to brown with a yellow glow	−/+	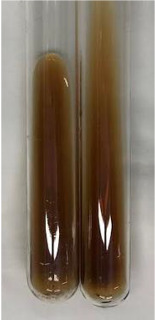	The colour of the solution changes to brown-orange	−	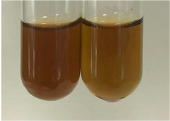	The appearance of a jelly-like consistency	−	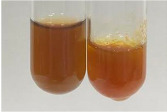
**To F**	Brown-orange colour of the solution; Brown-orange-yellow colour of the solution	−/+	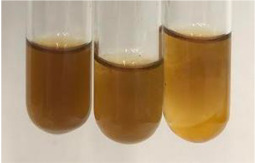	The colour of the solution changes to brown with a yellow glow	−/+	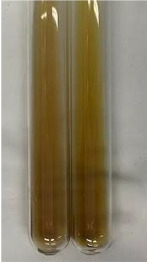	The colour of the solution changes to yellow	+	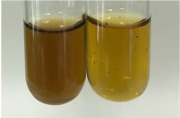	The colour of the solution changes to orange-red	−	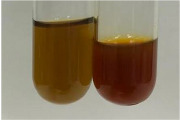
**To L**	Brown colour of the solution; Orange colour of the solution and precipitation of a brown precipitate	−/+	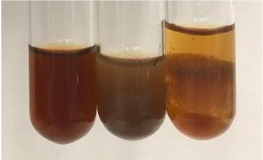	The colour of the solution changes to brown with a yellow glow	−/+	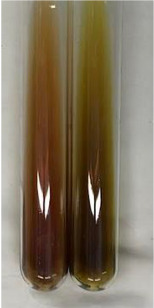	The colour of the solution changes to amber	−	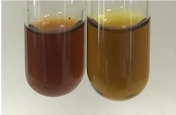	No changes were observed	−	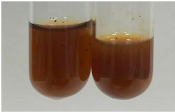
**To R**	Lemon colour of the solution; Lemon colour of the solution and precipitation of a yellow precipitate	−/+	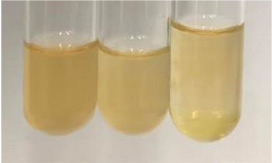	The colour of the solution changes to yellow	+	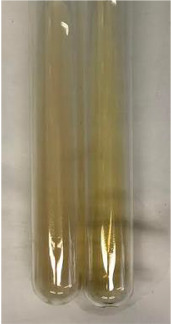	The colour of the solution changes to yellow	+	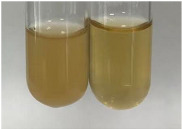	The colour of the solution changes to an intense orange	−	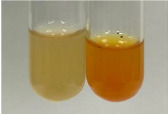
**Tp F**	Orange-yellow colour of the solution; Orange-yellow colour of the solution	−/+	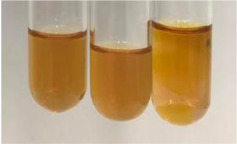	The colour of the solution changes to orange-yellow	−/+	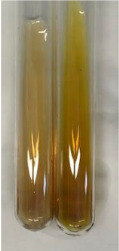	The colour of the solution changes to yellow	+	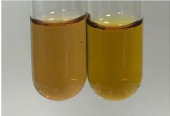	The colour of the solution changes to orange	−	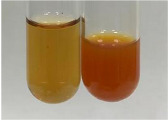
**Ur L**	Olive green colour of the solution; Orange colour of solution and precipitation	−/+	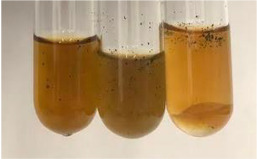	No changes were observed	−	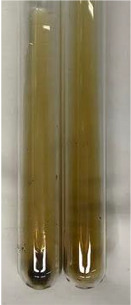	The colour of the solution changes to yellow-green	−/+	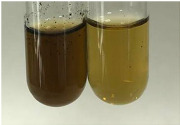	The colour of the solution changes to orange-brown	−	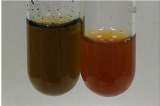
**Ur R**	Yellow colour of the solution; Yellow colour of the solution	−/+	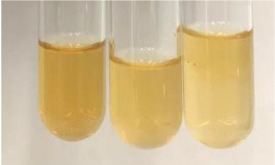	No changes were observed	−	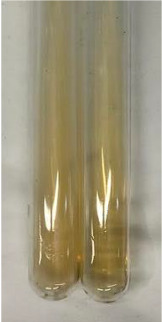	The colour of the solution changes to lemon	−/+	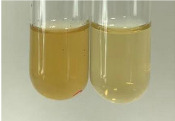	The colour of the solution changes to an intense orange	−	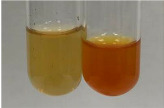
**Vo R**	Orange-brown colour of the solution; Brown-yellow colour of the solution	−/+	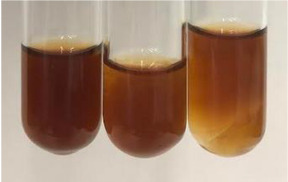	No changes were observed	−	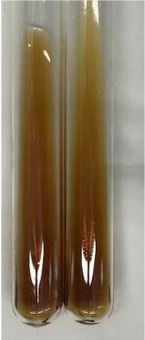	The colour of the solution changes to orange	−	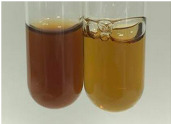	Changing the colour of the solution to a darker one	−	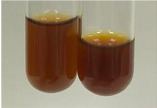

+—present; −—not present; −/+—not obvious. Abbreviations: Alv L—aloe leaves; Am Fr—black chokeberry fruits; Arv H—common mugwort herb; Bv R—beetroot roots; Co F—common marigold flowers; Ea H—field horsetail herb; Ep F—purple coneflower flowers; Ep L—purple coneflower leaves; Hp H—St. John’s wort herb; Hr Fr—sea-buckthorn fruits; Lc S—red lentil seeds; Mc F—chamomile flowers; Ob H—basil herb; Pm H—broadleaf plantain herb; Poa H—common knotgrass herb; Ps S—pea seeds; Pta L—common bracken leaves; Sg L—giant goldenrod leaves; So R—comfrey roots; To F—common dandelion flowers; To L—common dandelion leaves; To R—common dandelion roots; Tp F—red clover flowers; Ur L—nettle leaves; Ur R—nettle roots; Vo R—valerian roots.

**Table 7:** The results of H_2_SO_4_ test, HCl test, ammonia test, NaOH test (QNO—quinones, QNI—quinines).

Method	H_2_SO_4_ Test			HCl Test			Ammonia Test			NaOH Test		
Extract	Observation	QNO	Photo	Observation	QNO	Photo	Observation	QNO	Photo	Observation	QNI	Photo
**Alv L**	A change in the colour of the solution to dark brown	−	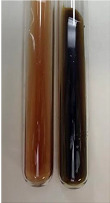	A change in the colour of the solution to yellow with an admixture of orange	−	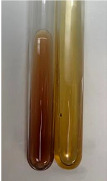	The colour of the solution changes to orange with a yellow glow	−	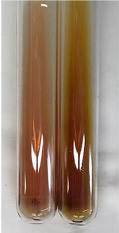	The colour of the solution changes to yellow-orange	−	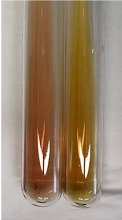
**Am Fr**	A colour change of the solution to dark red	+	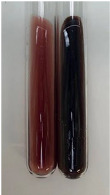	A colour change of the solution to a vivid red	−	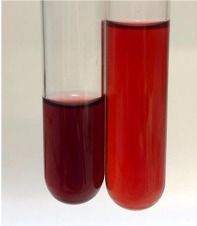	The colour of the solution changes to brown	−	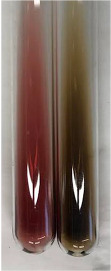	The colour of the solution changes to brown with a yellow glow	−	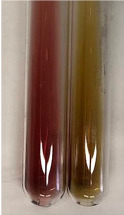
**Arv H**	A change in the colour of the solution to dark brown	−	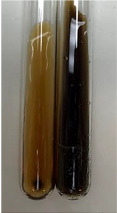	A change in the colour of the solution to orange with an admixture of yellow	−	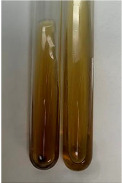	The colour of the solution changes to olive green	−	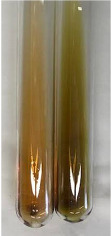	The colour of the solution changes to yellow-orange	−	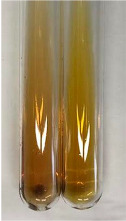
**Bv R**	A change in the colour of the solution to dark brown	−	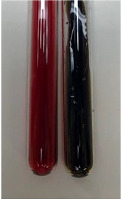	A colour change of the solution to maroon	−	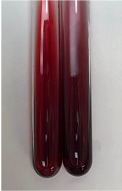	The colour of the solution changes to red with a yellow glow	−	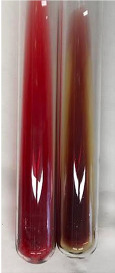	The colour of the solution changes to yellow-orange	−	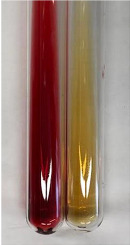
**Co F**	A change in the colour of the solution to dark brown	−	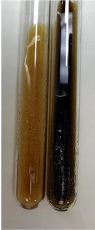	A colour change of the solution to orange with precipitation	−/+	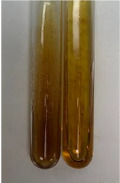	The colour of the solution changes to orange with a yellow glow	−	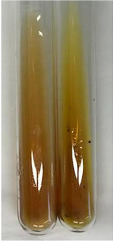	The colour of the solution changes to yellow-orange	−	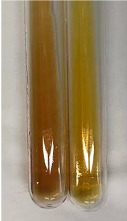
**Ea H**	A change in the colour of the solution to brown	−	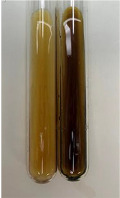	A colour change of the solution to yellow	−	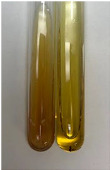	The colour of the solution changes to brown-orange with a yellow glow	−	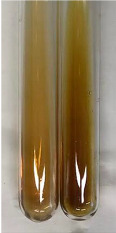	The colour of the solution changes to orange with a yellow glow	−	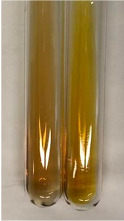
**Ep F**	A change in the colour of the solution to dirty brown and the formation of a fine precipitate	−	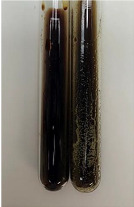	The colour of the solution turned yellow and the precipitation of a brown precipitate	−/+	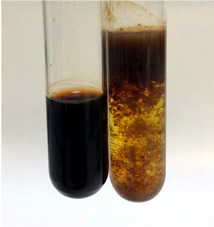	The colour of the solution changes to brown with a yellow glow	−	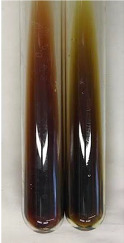	The colour of the solution changes to brown with a yellow glow	−	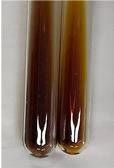
**Ep L**	A change in the colour of the solution to brown	−	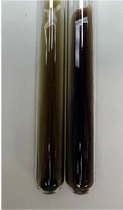	A colour change of the solution to dirty yellow	−	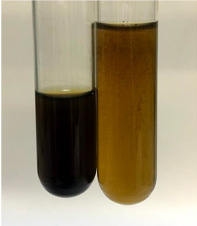	The colour of the solution changes to brown with a yellow glow	−	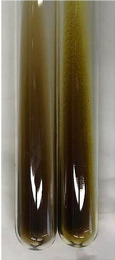	The colour of the solution changes to orange with a yellow glow	−	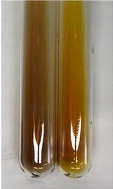
**Hp H**	A change in the colour of the solution to dark brown	−	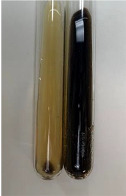	A colour change of the solution to orange	−	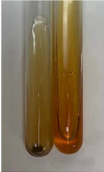	The colour of the solution changes to orange with a yellow glow	−	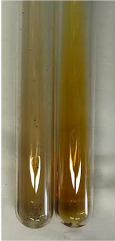	The colour of the solution changes to yellow	−	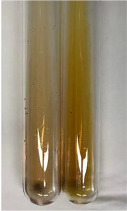
**Hr Fr**	A change in the colour of the solution to brown	−	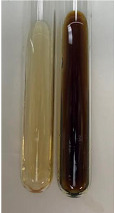	A colour change of the solution to an intense yellow	−	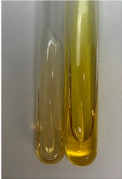	The colour of the solution changes to neon yellow	−	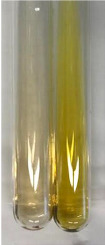	The colour of the solution changes to neon new yellow	−	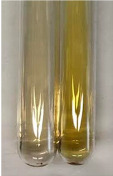
**Lc S**	A change in the colour of the solution to brown	−	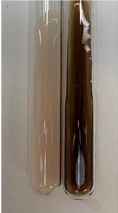	A change in the colour of the solution to lemon	−	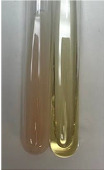	The colour of the solution changes to dirty yellow	−	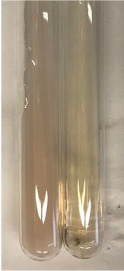	The colour of the solution changes to pale yellow	−	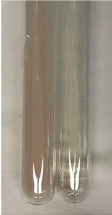
**Mc F**	A change in the colour of the solution to dark brown	−	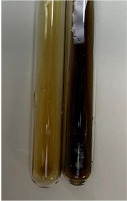	A colour change of the solution to yellow	−	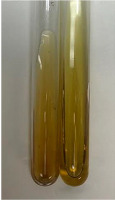	The colour of the solution changes to orange-yellow	−	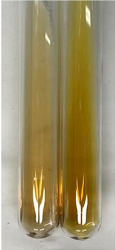	The colour of the solution changes to yellow	−	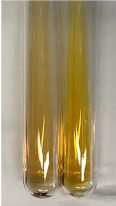
**Ob H**	A change in the colour of the solution to dark brown	−	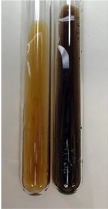	A colour change of the solution to orange-yellow	−	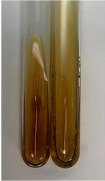	The colour of the solution changes to brown with a yellow glow	−	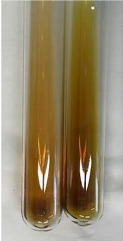	The colour of the solution changes to yellow-orange	−	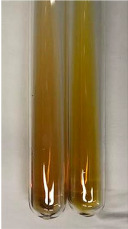
**Pm H**	A change in the colour of the solution to dark brown	−	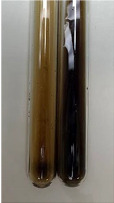	A colour change of the solution to orange-yellow	−	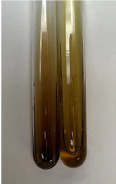	The colour of the solution changes to brown with a yellow glow	−	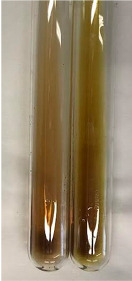	The colour of the solution changes to yellow-orange	−	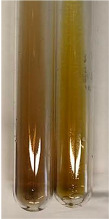
**Poa H**	A colour change of the solution to amber	−	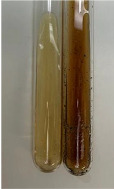	A colour change of the solution to an intense yellow	−	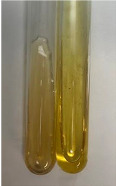	The colour of the solution changes to an intense yellow	−	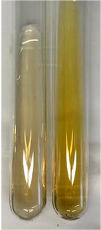	The colour of the solution changes to lemon-yellow	−	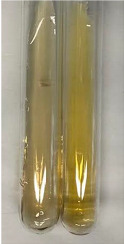
**Ps S**	A change in the colour of the solution to brown	−	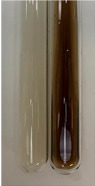	A change in the colour of the solution to a delicate lemon	−	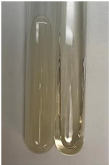	The colour of the solution changes to light yellow	−	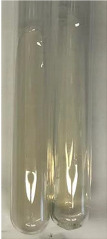	Change of colour of the solution to colourless-lemon	−	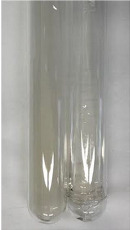
**Pta L**	A change in the colour of the solution to dark brown	−	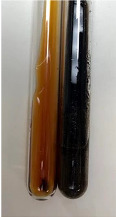	A colour change of the solution to dirty yellow	−	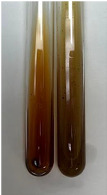	The colour of the solution changes to orange	−	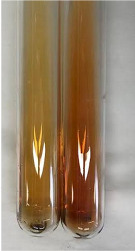	The colour of the solution changes to dark orange	−	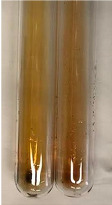
**Sg L**	A change in the colour of the solution to dark brown	−	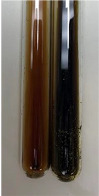	A change in the colour of the solution to orange with an admixture of yellow	−	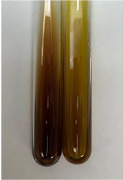	The colour of the solution changes to brown with a yellow glow	−	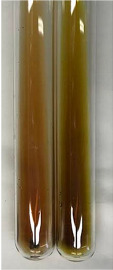	The colour of the solution changes to yellow-orange	−	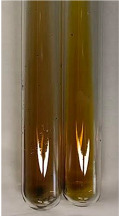
**So R**	A change in the colour of the solution to dark brown	−	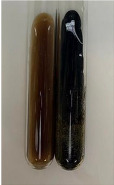	A colour change of the solution to colourless with precipitation of an orange precipitate	−/+	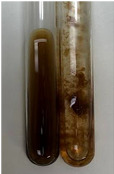	No changes were observed	−	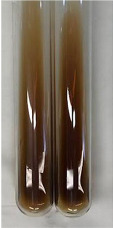	The colour of the solution changes to yellow-orange	−	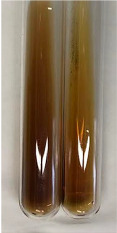
**To F**	A change in the colour of the solution to brown	−	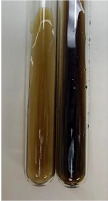	A colour change of the solution to yellow-orange	−	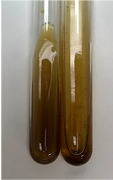	The colour of the solution changes to an intense yellow	−	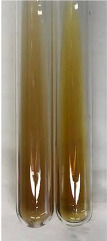	The colour of the solution changes to orange with a yellow glow	−	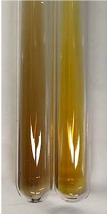
**To L**	A change in the colour of the solution to dark brown	−	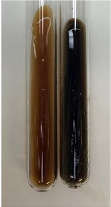	A colour change of the solution to yellow-orange with precipitation	−/+	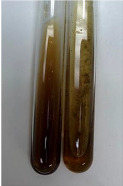	The colour of the solution changes to brown with a yellow glow	−	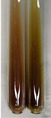	The colour of the solution changes to orange with a yellow glow	−	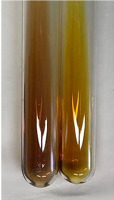
**To R**	A colour change of the solution to black-brown	−	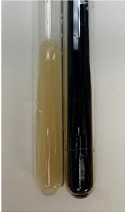	A change in the colour of the solution to a soft orange	−	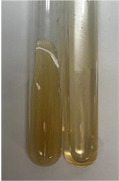	The colour of the solution changes to yellow	−	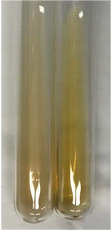	The colour of the solution changes to yellow	−	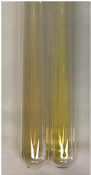
**Tp F**	A change in the colour of the solution to dark brown	−	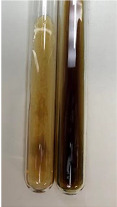	A colour change of the solution to orange-yellow	−	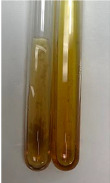	The colour of the solution changes to orange-yellow	−	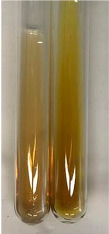	The colour of the solution changes to yellow	−	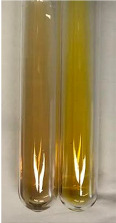
**Ur L**	A colour change of the solution to bright orange	−	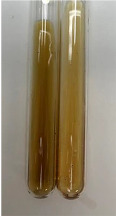	A colour change of the solution to a dirty orange	−	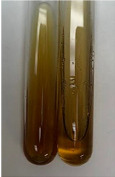	No changes were observed	−	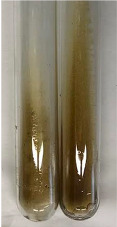	The colour of the solution changes to yellow	−	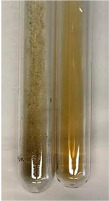
**Ur R**	A change in the colour of the solution to an intense orange with an admixture of yellow	−/+	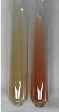	A change in the colour of the solution to lemon	−	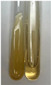	No changes were observed	−	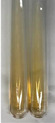	The colour of the solution changes to lemon	−	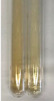
**Vo R**	A change in the colour of the solution to dark brown	−	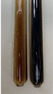	A change in the colour of the solution to orange with an admixture of brown	−	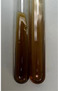	No changes were observed	−	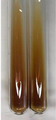	The colour of the solution changes to yellow-orange	−	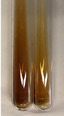

+—present; −—not present; −/+—not obvious. Abbreviations: Alv L—aloe leaves; Am Fr—black chokeberry fruits; Arv H—common mugwort herb; Bv R—beetroot roots; Co F—common marigold flowers; Ea H—field horsetail herb; Ep F—purple coneflower flowers; Ep L—purple coneflower leaves; Hp H—St. John’s wort herb; Hr Fr—sea-buckthorn fruits; Lc S—red lentil seeds; Mc F—chamomile flowers; Ob H—basil herb; Pm H—broadleaf plantain herb; Poa H—common knotgrass herb; Ps S—pea seeds; Pta L—common bracken leaves; Sg L—giant goldenrod leaves; So R—comfrey roots; To F—common dandelion flowers; To L—common dandelion leaves; To R—common dandelion roots; Tp F—red clover flowers; Ur L—nettle leaves; Ur R—nettle roots; Vo R—valerian roots.

**Table 8:** The presence of cardiac glycosides and resin in botanical extracts—Baljet test and acetone test.

Method	Baljet Test			Acetone Test		
Extract	Observation	CGS	Photo	Observation	RN	Photo
**Alv L**	The colour of the solution changes to orange with a yellow glow	−	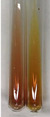	Formation of 2 phases: orange with precipitate and orange	+	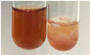
**Am Fr**	The colour of the solution changes to brown-orange with a yellow glow	−	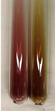	Formation of 2 phases: light red and cloudy red	+	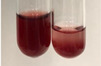
**Arv H**	The colour of the solution changes to brown-olive	−	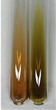	Formation of 2 phases: orange and cloudy orange	+	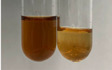
**Bv R**	The colour of the solution changes to red	−	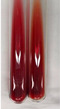	A change in the colour shade of the solution	−	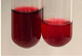
**Co F**	The colour of the solution changes to brown-orange with a yellow glow	−	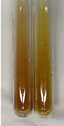	Formation of 2 phases: cloudy orange and orange	+	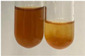
**Ea H**	The colour of the solution changes to orange with a yellow glow	−/+	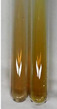	Formation of 2 phases: turbid yellow and orange	+	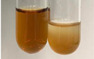
**Ep F**	The appearance of a yellow glow on the walls of the tube	−	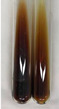	Formation of 2 phases: precipitate and a clear solution	−/+	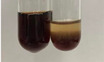
**Ep L**	The colour of the solution changes to brown with a yellow glow	−	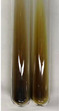	Formation of 3 phases: clear solution, precipitate and orange	−/+	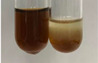
**Hp H**	The colour of the solution changes to yellow	−	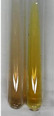	Formation of 2 phases: pale orange and pale yellow	−	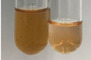
**Hr Fr**	The colour of the solution changes to intense yellow	−	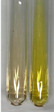	A colour change to light yellow	−	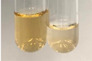
**Lc S**	The colour of the solution changes to yellow	−	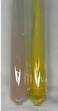	Formation of 2 phases: cloudy-orange-pink and pink	+	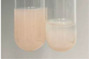
**Mc F**	The colour of the solution changes to yellow	−	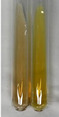	Formation of 2 phases: clear lemon and yellow	−	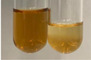
**Ob H**	The colour of the solution changes to orange with a yellow glow	−/+	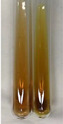	Formation of 2 phases: yellow with precipitate and orange	+	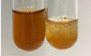
**Pm H**	The colour of the solution changes to brown-orange with a yellow glow	−	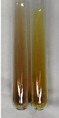	Formation of 2 phases: cloudy yellow and cloudy orange	+	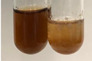
**Poa H**	The colour of the solution changes to yellow	−	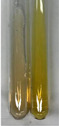	A change in the colour of the solution to a light lemon colour	−	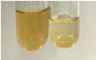
**Ps S**	The colour of the solution changes to yellow	−	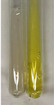	Formation of 2 phases: cloudy white and white	+	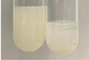
**Pta L**	The colour of the solution changes to orange with a yellow glow	−/+	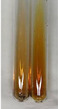	Formation of 2 phases: yellow and cloudy yellow-orange	+	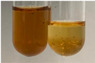
**Sg L**	The colour of the solution changes to brown with a yellow glow	−	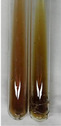	Formation of 2 phases: turbid yellow and orange	+	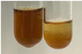
**So R**	The colour of the solution changes to brown with a yellow glow	−	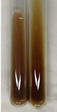	Formation of 2 phases: cloudy orange and amber-orange	+	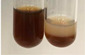
**To F**	The colour of the solution changes to yellow-orange	−/+	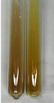	Formation of 2 phases: pale yellow and cloudy orange	+	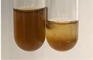
**To L**	The colour of the solution changes to brown with a yellow glow	−	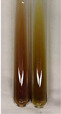	A change in the colour of the solution to orange	−	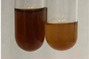
**To R**	The colour of the solution changes to intense yellow	−	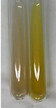	A change in the colour of the solution to lemon	−	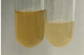
**Tp F**	The colour of the solution changes to yellow	−	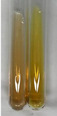	Formation of 2 phases: clear lemon and yellow	−	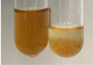
**Ur L**	The colour of the solution changes to orange with a yellow glow	−	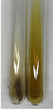	A colour change of the solution to brown-olive	−	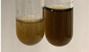
**Ur R**	The colour of the solution changes to intense yellow	−	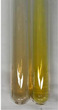	Formation of 2 phases: turbid yellow and yellow	+	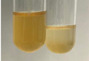
**Vo R**	The colour of the solution changes to brown with a yellow glow	−	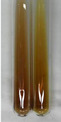	Formation of 2 phases: cloudy orange and amber-orange	+	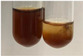

+—present; −—not present; −/+—not obvious. Abbreviations: Alv L—aloe leaves; Am Fr—black chokeberry fruits; Arv H—common mugwort herb; Bv R—beetroot roots; Co F—common marigold flowers; Ea H—field horsetail herb; Ep F—purple coneflower flowers; Ep L—purple coneflower leaves; Hp H—St. John’s wort herb; Hr Fr—sea-buckthorn fruits; Lc S—red lentil seeds; Mc F—chamomile flowers; Ob H—basil herb; Pm H—broadleaf plantain herb; Poa H—common knotgrass herb; Ps S—pea seeds; Pta L—common bracken leaves; Sg L—giant goldenrod leaves; So R—comfrey roots; To F—common dandelion flowers; To L—common dandelion leaves; To R—common dandelion roots; Tp F—red clover flowers; Ur L—nettle leaves; Ur R—nettle roots; Vo R—valerian roots.

**Table 9:** The presence of glycosides in botanical extracts—Keller–Killiani test, Borntrager’s tests (1), Borntrager’s tests (2), Molisch’s test (additionally: sugars) (GS—glycosides, CGS—cardiac glycosides, CYGS—cyanogenic glycosides, SG—sugars).

Method	Keller–Killiani Test			Borntrager’s Tests (1)			Borntrager’s Tests (2)				Molisch’s Test			
Extract	Observation	CGS	Photo	Observation	CYGS	Photo	Observation	GS	SG	Photo	Observation	GS	SG	Photo
**Alv L**	Formation of 3 phases: olive green, orange and brown-red	−	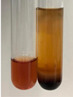	Formation of 2 phases: yellow and colourless	−	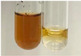	Formation of 2 phases: brown- orange with a yellow glow and colourless	−	−	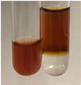	Formation of 3 phases: cloudy orange, violet-red and colourless	+	+	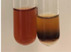
**Am Fr**	Formation of 3 phases: red, raspberry, and black	−	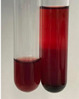	Formation of 2 phases: orange-pink and colourless	−	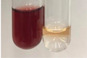	Formation of 2 phases: brown- orange with a yellow glow and colourless	−	−	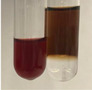	Appearance of a raspberry-coloured phase in the upper part of the tube and a black phase in the lower part	−	−	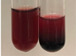
**Arv H**	Formation of 3 phases: brown, orange, and dark brown	−	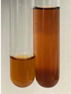	Formation of 2 phases: yellow and colourless	−	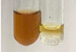	Formation of 2 phases: olive green with a glow of yellow and colourless	−	−	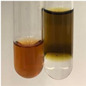	Formation of 3 phases: cloudy-orange, violet-red and colourless	+	+	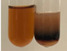
**Bv R**	Formation of 3 phases: red, brown-red, and bloody	−/+	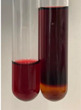	Formation of 2 phases: yellow and colourless	−	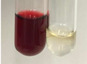	Formation of 2 phases: orange with a yellow glow and colourless	−	−	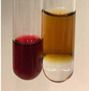	Formation of 2 phases: dark red and green	−	−	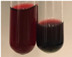
**Co F**	Formation of 4 phases: black, brown, orange, and dark red	−/+	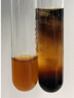	Formation of 2 phases: yellow and colourless	−	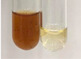	Formation of 2 phases: orange with a yellow glow and colourless	−	−	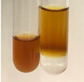	Formation of 3 phases: amber, violet-red and colourless	+	+	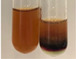
**Ea H**	Formation of 3 phases: olive-brown, yellow, and orange-brown	−	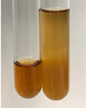	Formation of 2 phases: light yellow and colourless	−	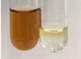	Formation of 2 phases: brown- orange with a yellow glow and colourless	−	−	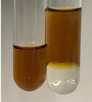	Formation of 3 phases: yellow, red-violet and colourless	+	+	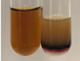
**Ep F**	Formation of 3 phases: brown, brick red, and brown	−/+	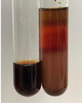	Colourless solution	−	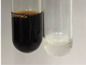	Formation of 2 phases: black with a yellow glow and colourless	−	−	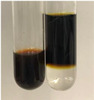	The appearance of a brick-red precipitate in the upper part of the solution and a brown solution in the bottom of the test tube	−	−	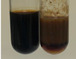
**Ep L**	Formation of 3 phases: olive, orange, and green-brown	−	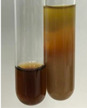	Formation of 2 phases: yellow and colourless	−	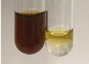	Formation of 2 phases: brown with a yellow glow and colourless	−	−	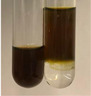	The appearance of a cappuccino-coloured phase in the upper part of the tube and a brown solution in the lower part	−	−	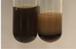
**Hp H**	Formation of 3 phases: orange-red, red, and black-red	−/+	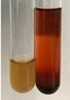	Formation of 2 phases: orange-pink and colourless	−	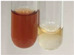	Formation of 2 phases: orange with a yellow glow and colourless	−	−	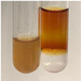	Appearance of a cloudy-orange phase with a slight precipitate and a violet-red phase	−	−	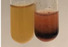
**Hr Fr**	Formation of 3 phases: dirty yellow, orange, and brown-red	−	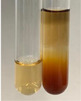	Formation of 2 phases: lemon and colourless	−	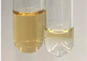	Formation of 2 phases: yellow and colourless	−	−	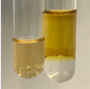	The appearance of a yellow phase in the upper part of the test tube, and a violet-brown-red ring below it	−	−	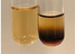
**Lc S**	Formation of 3 phases: pale yellow, soft pink, and brown-red	−	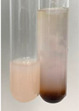	Colourless solution	−	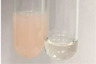	Formation of 2 phases: lemon and colourless	−	−	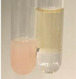	Formation of 3 phases: cloudy white, red-violet and colourless	+	+	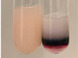
**Mc F**	Formation of 3 phases: brown, dirty yellow, and red-brown	−	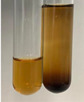	Formation of 2 phases: yellow and colourless	−	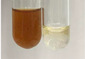	Formation of 2 phases: orange with a yellow glow and colourless	−	−	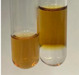	Formation of 3 phases: cloudy-yellow, violet-red and beige	+	+	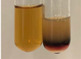
**Ob H**	Formation of 3 phases: brown, orange, and dark brown	−	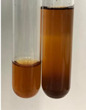	Formation of 2 phases: yellow and colourless	−	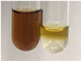	Formation of 2 phases: brown with a yellow glow and colourless	−	−	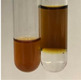	Formation of 3 phases: cloudy-yellow-brown, violet-brown and green	+	+	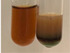
**Pm H**	Formation of 2 phases: brown and black	−	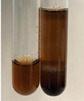	Formation of 2 phases: yellow and colourless	−	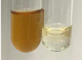	Formation of 2 phases: orange with a yellow glow and colourless	−	−	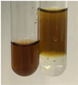	Formation of 2 phases: cloudy olive-brown and violet-red	−	−	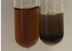
**Poa H**	Formation of 3 phases: pale yellow, yellow, and amber	−	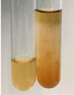	Formation of 2 phases: lemon and colourless	−	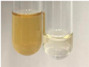	Formation of 2 phases: orange-yellow and colourless	−	−	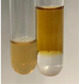	Formation of 3 phases: light brown, dark brown and green	+	+	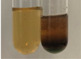
**Ps S**	Formation of 3 phases: pale yellow, soft pink, and brown-red	−	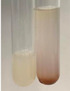	Colourless solution	−	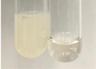	Formation of 2 phases: pale lemon and colourless	−	−	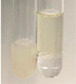	Formation of 3 phases: cloudy white, red-violet and colourless	+	+	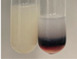
**Pta L**	Formation of 3 phases: brown, orange, and red-brown	−	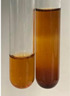	Formation of 2 phases: orange-pink and colourless	−	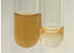	Formation of 2 phases: orange-red and colourless	−	−	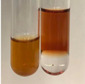	Formation of 2 phases: cloudy-yellow and violet-brown	−	−	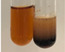
**Sg L**	Formation of 4 phases: brown, orange, olive green, and black	−/+	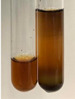	Formation of 2 phases: yellow and colourless	−	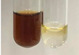	Formation of 2 phases: brown with a yellow glow and colourless	−	−	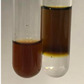	Formation of 3 phases: cloudy- yellow, violet-brown and violet-red	+	+	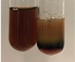
**So R**	Formation of 3 phases: brown, orange, and black	−/+	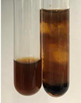	Formation of 2 phases: yellow and colourless	−	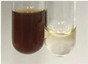	Formation of 2 phases: brown with a yellow glow and colourless	−	−	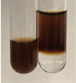	Formation of 3 phases: brick-orange, violet-red and colourless	+	+	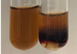
**To F**	Formation of 3 phases: olive, orange, and brown-red	−/+	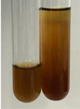	Formation of 2 phases: yellow and colourless	−	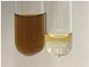	Formation of 2 phases: orange and colourless	−	−	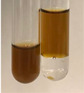	Formation of 2 phases: dirty brown and black-brown	−	−	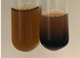
**To L**	Formation of 3 phases: orange-brown, red, and dark brown	+	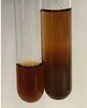	Formation of 2 phases: yellow and colourless	−	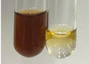	Formation of 2 phases: brown with a yellow glow and colourless	−	−	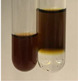	Formation of 2 phases: brown and violet-red-brown	−	−	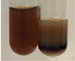
**To R**	Formation of 3 phases: pale yellow, yellow, and blood- hundred red	−	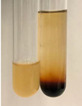	Formation of 2 phases: yellow and colourless	−	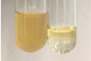	Formation of 2 phases: yellow and colourless	−	−	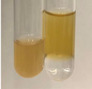	Formation of 2 phases: cloudy yellow and purple	−	−	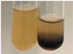
**Tp F**	Formation of 3 phases: brown, orange, and red-brown	−	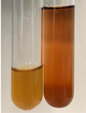	Formation of 2 phases: yellow and colourless	−	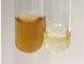	Formation of 2 phases: orange and colourless	−	−	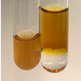	Formation of 3 phases: cloudy-orange, violet-red and colourless	+	+	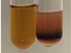
**Ur L**	Formation of 4 phases: brown, brown-red, orange, and yellow	+	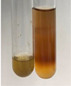	Formation of 2 phases: light orange and colourless	−	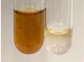	Formation of 2 phases: olive-brown and colourless	−	−	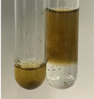	Formation of 3 phases: light brown, pink-purple and green	+	+	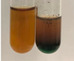
**Ur R**	Formation of 3 phases: lemon, yellow, and orange-yellow	−	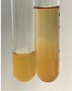	Formation of 2 phases: light lemon and colourless	−	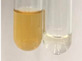	Formation of 2 phases: lemon and colourless	−	−	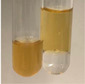	Formation of 3 phases: yellow, purple-brown and green	+	+	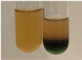
**Vo R**	Formation of 3 phases: orange-amber, maroon, and brown-red	+	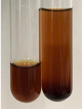	Formation of 2 phases: yellow and colourless	−	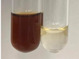	Formation of 2 phases: brown with a yellow glow and colourless	−	−	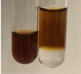	Formation of 2 phases: cloudy brown and purple-red	−	−	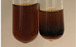

+—present; −—not present; −/+—not obvious. Abbreviations: Alv L—aloe leaves; Am Fr—black chokeberry fruits; Arv H—common mugwort herb; Bv R—beetroot roots; Co F—common marigold flowers; Ea H—field horsetail herb; Ep F—purple coneflower flowers; Ep L—purple coneflower leaves; Hp H—St. John’s wort herb; Hr Fr—sea-buckthorn fruits; Lc S—red lentil seeds; Mc F—chamomile flowers; Ob H—basil herb; Pm H—broadleaf plantain herb; Poa H—common knotgrass herb; Ps S—pea seeds; Pta L—common bracken leaves; Sg L—giant goldenrod leaves; So R—comfrey roots; To F—common dandelion flowers; To L—common dandelion leaves; To R—common dandelion roots; Tp F—red clover flowers; Ur L—nettle leaves; Ur R—nettle roots; Vo R—valerian roots.

**Table 10:** The presence of sugars in botanical extracts—Fehling’s test, Benedict’s test, Selwinoff’s test, Barfoed’s test (SG—sugars).

Method	Fehling’s Test			Benedict’s Test			Selwinoff’s Test			Barfoed’s Test		
Extract	Observation	SG	Photo	Observation	SG	Photo	Observation	SG	Photo	Observation	SG	Photo
**Alv L**	A colour change to brick-brown	−	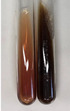	Intense olive colour; Orange-brick colour + red precipitate	+	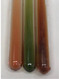	The colour of the solution changes to orange	−	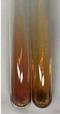	Green colour of the solution; Dark green colour of the solution	−	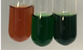
**Am Fr**	A colour change to dark amber	−	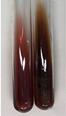	Intense green colour + fine precipitate; Olive colour + red precipitate	+	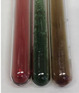	The colour of the solution changes to bright red	+	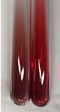	Green-blue solution + precipitation; Green-blue solution + black precipitate	−	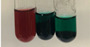
**Arv H**	A colour change to dark green	−	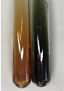	Intense colouring; Olive colour + brick red precipitate	+	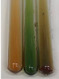	The colour of the solution changes to brown-orange	−	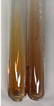	Green colour + brick red precipitate; Green colour + brick red precipitate	+	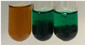
**Bv R**	A colour change to dirty brown	−	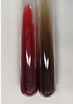	Olive colour; Cloudy orange solution + orange precipitate	+	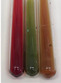	The colour of the solution changes to orange-brick	+	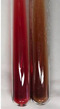	Dark green colour; Green solution + precipitate	−	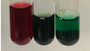
**Co F**	A colour change to dark yellow-orange	−	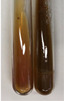	Green-yellow colour; Orange-yellow colour + red-orange precipitate	+	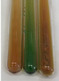	The colour of the solution changes to orange	−	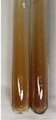	Green-cloudy colour of the solution; Green colour of the solution + precipitate	−	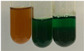
**Ea H**	A colour change to green with a dark maroon glow at the bottom	−	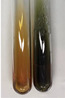	Intense green solution; Orange solution + orange precipitate	+	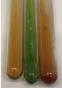	No changes were observed	−	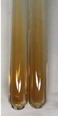	Dark green colour + slight precipitate; Green colour of the solution + precipitate	−	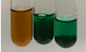
**Ep F**	A colour change to dark brown	−	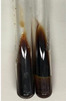	Dirty olive green; Brown-orange solution + orange precipitate	+	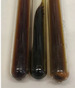	The colour of the solution changes to orange	−	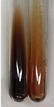	Cloudy olive solution; Cloudy olive solution	−	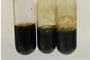
**Ep L**	A colour change to dark green	−	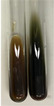	Intense green solution; Olive green + orange precipitate	+	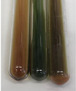	The colour of the solution changes to orange	−	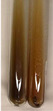	Cloudy green solution + fine precipitate; Cloudy green solution + fine precipitate	−	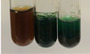
**Hp H**	A colour change to brick red	−	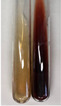	Green solution; Brown-orange colour + brick red precipitate	+	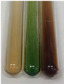	The colour of the solution changes to red-orange	+	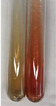	Intense green colour of the precipitate solution; Intense green colour + brick-hundred-brown precipitate	−	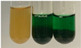
**Hr Fr**	A colour change to intense green with a dark maroon glow at the bottom of the tube	−	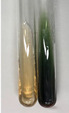	Intense light green solution; Light olive green + reddish-brown precipitate	+	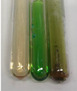	The colour of the solution changes to a vivid yellow-orange	−	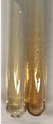	Green colour + precipitation of a delicate precipitate; Green colour + precipitation	−	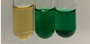
**Lc S**	A colour change to navy blue	−	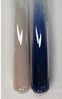	Bright turquoise solution; Green solution	−	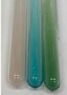	The colour of the solution changes to cloudy- colourless	−	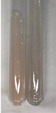	Light blue solution; Blue colour of the solution + white precipitate	−	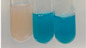
**Mc F**	A colour change to green	−	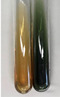	Intense green; Cloudy orange-brown solution + orange precipitate	+	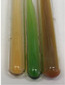	The colour of the solution changes to olive green	−	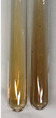	Green colour of the solution; Green colour of the solution + green precipitate	−	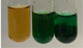
**Ob H**	A colour change to green	−	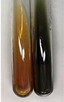	Intense green; Olive colour + brick red precipitate	+	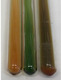	The colour of the solution changes to brown-orange	−	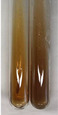	Blue-green colour + dark green precipitate; Green-turquoise colour + brick-hundred-brown precipitate	−	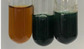
**Pm H**	A colour change to green with a dark maroon glow at the bottom	−	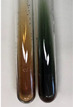	Intense green; Olive colour + brick red precipitate	+	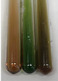	The colour of the solution changes to brown	−	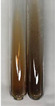	Dark green solution + precipitate; Dark green solution + dark precipitate	−	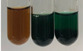
**Poa H**	A colour change to intense green	−	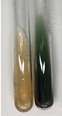	Intense green; Olive colour + red precipitate	+	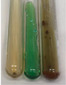	The colour of the solution changes to orange with the formation of a precipitate	−	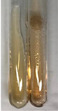	Green colour of the solution + precipitation; Green colour of the solution + precipitation	−	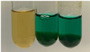
**Ps S**	A colour change to blue with a dark green glow	−	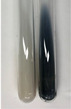	Light turquoise solution; Intense green	−	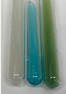	Change of colour of the solution to cloudy powder pink	−	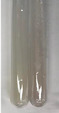	Blue colour of the solution; Gelatinous, blue consistency of the solution	−	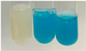
**Pta L**	A colour change to bloody red	−	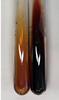	Green colouration; Brown-orange colour + red precipitate	+	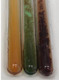	The colour of the solution changes to orange	−	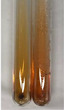	Green colour of the solution + brick-red precipitate; Green-turquoise colour + brick red precipitate	+	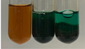
**Sg L**	A colour change to a dark olive green	−	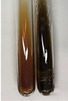	Intense green; Olive-brown colour + red precipitate	+	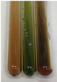	The colour of the solution changes to brown with the formation of a precipitate	−	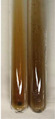	Dark green colour + precipitate; Green colour + dark olive precipitate	−	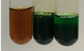
**So R**	A colour change to a dirty olive green	−	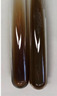	The appearance of a blue-green colour; Olive-orange solution + orange precipitate	+	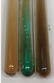	The colour of the solution changes to orange with the formation of a precipitate	−	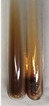	Turquoise colour, black precipitate, gelatinous solution form; Turquoise colour + black precipitate	−	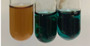
**To F**	A colour change to orange-amber	−	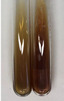	Intense green; Cloudy orange solution + orange precipitate	+	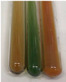	The colour of the solution changes to orange-amber	−	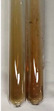	Green colour of the solution + precipitation of a green precipitate; Green colour of the solution green-brown precipitate	−	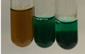
**To L**	A colour change to a dirty olive green	−	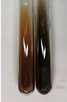	Intense green; Cloudy orange solution + orange precipitate	+	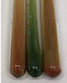	The colour of the solution changes to orange-amber	−	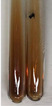	Cloudy green solution + fine precipitate; Green solution + green precipitate	−	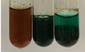
**To R**	A colour change to brick red	−	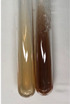	Green + cloudy colour of the solution + precipitate	+	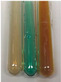	The colour of the solution changes to orange	−	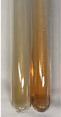	Turquoise solution + white precipitate; Turquoise colour + brick red precipitate	+	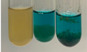
**Tp F**	A colour change to green	−	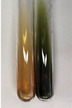	Intense green; Yellow-brown colour + red precipitate	+	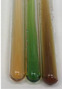	The colour of the solution changes to orange-amber	−	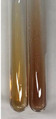	Green/cloudy solution; Green solution + precipitate	−	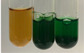
**Ur L**	A colour change to intense green	−	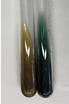	Bottle green; Olive colour + red-orange precipitate	+	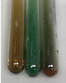	The colour of the solution changes to orange-amber	−	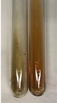	Green colour of the solution + precipitation of a delicate precipitate; Turquoise/green colour + precipitation	−	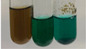
**Ur R**	A colour change to blue with a dark maroon glow at the bottom	−	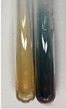	Green solution; Cloudy orange solution + orange precipitate	+	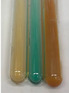	The colour of the solution changes to orange	−	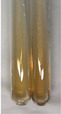	Turquoise solution + white precipitate; Turquoise colour + white-brick precipitate	−	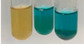
**Vo R**	A colour change to a dirty olive green	−	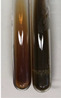	Intense green; Dirty olive solution + precipitate	+	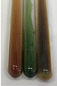	No changes were observed	−	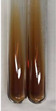	Green colour of the solution + slight green precipitate; Green colour of the solution + green precipitate	−	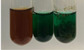

+—present; −—not present; −/+—not obvious. Abbreviations: Alv L—aloe leaves; Am Fr—black chokeberry fruits; Arv H—common mugwort herb; Bv R—beetroot roots; Co F—common marigold flowers; Ea H—field horsetail herb; Ep F—purple coneflower flowers; Ep L—purple coneflower leaves; Hp H—St. John’s wort herb; Hr Fr—sea-buckthorn fruits; Lc S—red lentil seeds; Mc F—chamomile flowers; Ob H—basil herb; Pm H—broadleaf plantain herb; Poa H—common knotgrass herb; Ps S—pea seeds; Pta L—common bracken leaves; Sg L—giant goldenrod leaves; So R—comfrey roots; To F—common dandelion flowers; To L—common dandelion leaves; To R—common dandelion roots; Tp F—red clover flowers; Ur L—nettle leaves; Ur R—nettle roots; Vo R—valerian roots.

**Table 11:** The antioxidant activity of botanical extracts—DPPH assay, ABTS assay, FRAP assay.

Method	Antioxidant Activity—DDPH	Antioxidant Activity—ABTS	Antioxidant Activity—FRAP	Antioxidant Activity—DDPH	Antioxidant Activity—ABTS
Extract	µM Trolox·mL^−1^			Inhibition Ratio (%)	
**Alv L**	0.73 ± 0.09	0.86 ± 0.14	1.38 ± 0.04	12.71 ± 0.02	0.74 ± 0.12
**Am Fr**	3.99 ± 0.14	10.94 ± 1.63	8.73 ± 0.11	6.60 ± 0.00	0.91 ± 0.14
**Arv H**	1.60 ± 0.02	3.45 ± 0.37	5.60 ± 0.01	28.12 ± 0.00	2.95 ± 0.32
**Bv R**	0.83 ± 0.01	3.17 ± 0.35	3.26 ± 0.06	14.11 ± 0.00	2.65 ± 0.30
**Co F**	0.97 ± 0.07	2.59 ± 0.28	3.31 ± 0.11	16.53 ± 0.01	2.16 ± 0.24
**Ea H**	0.72 ± 0.06	1.50 ± 0.22	2.58 ± 0.08	12.55 ± 0.01	1.25 ± 0.18
**Ep F**	4.23 ± 0.20	15.54 ± 1.41	12.41 ± 0.15	7.02 ± 0.00	1.29 ± 0.12
**Ep L**	1.58 ± 0.17	19.00 ± 1.41	15.28 ± 0.11	2.37 ± 0.00	1.58 ± 0.12
**Hp H**	4.55 ± 0.33	12.67 ± 0.81	8.90 ± 0.11	7.58 ± 0.01	1.08 ± 0.07
**Hr Fr**	1.78 ± 0.05	1.84 ± 0.16	3.64 ± 0.01	31.48 ± 0.01	1.53 ± 0.14
**Lc S**	0.14 ± 0.02	1.90 ± 0.14	0.40 ± 0.03	2.00 ± 0.00	1.58 ± 0.12
**Mc F**	0.92 ± 0.08	2.82 ± 0.43	3.27 ± 0.03	15.78 ± 0.01	2.36 ± 0.36
**Ob H**	2.48 ± 0.23	4.03 ± 0.81	11.74 ± 0.37	3.95 ± 0.00	0.34 ± 0.07
**Pm H**	1.79 ± 0.05	2.53 ± 0.33	5.56 ± 0.10	31.58 ± 0.01	2.17 ± 0.28
**Poa H**	0.65 ± 0.08	6.45 ± 0.43	2.70 ± 0.07	11.22 ± 0.01	5.37 ± 0.36
**Ps S**	0.15 ± 0.01	4.26 ± 0.33	0.43 ± 0.01	2.23 ± 0.00	3.55 ± 0.27
**Pta L**	9.57 ± 0.85	6.33 ± 0.81	20.25 ± 0.47	16.36 ± 0.01	0.54 ± 0.07
**Sg L**	3.41 ± 0.30	5.76 ± 0.81	9.25 ± 0.22	5.58 ± 0.01	0.48 ± 0.07
**So R**	1.37 ± 0.19	5.35 ± 0.61	5.76 ± 0.06	23.65 ± 0.03	4.47 ± 0.51
**To F**	0.90 ± 0.03	4.09 ± 0.29	2.71 ± 0.01	15.46 ± 0.01	3.42 ± 0.25
**To L**	2.61 ± 0.33	8.64 ± 1.41	6.62 ± 0.47	4.18 ± 0.01	0.72 ± 0.12
**To R**	0.43 ± 0.05	0.81 ± 0.16	0.97 ± 0.04	7.12 ± 0.01	0.67 ± 0.14
**Tp F**	1.21 ± 0.14	3.45 ± 0.51	4.51 ± 0.10	20.76 ± 0.02	2.89 ± 0.42
**Ur L**	0.14 ± 0.01	1.15 ± 0.08	1.09 ± 0.02	2.09 ± 0.00	0.96 ± 0.07
**Ur R**	0.15 ± 0.02	3.74 ± 0.22	0.82 ± 0.02	2.33 ± 0.00	3.12 ± 0.18
**Vo R**	0.76 ± 0.06	2.36 ± 0.35	2.43 ± 0.06	12.94 ± 0.01	1.97 ± 0.30

Abbreviations: Alv L—aloe leaves; Am Fr—black chokeberry fruits; Arv H—common mugwort herb; Bv R—beetroot roots; Co F—common marigold flowers; Ea H—field horsetail herb; Ep F—purple coneflower flowers; Ep L—purple coneflower leaves; Hp H—St. John’s wort herb; Hr Fr—sea-buckthorn fruits; Lc S—red lentil seeds; Mc F—chamomile flowers; Ob H—basil herb; Pm H—broadleaf plantain herb; Poa H—common knotgrass herb; Ps S—pea seeds; Pta L—common bracken leaves; Sg L—giant goldenrod leaves; So R—comfrey roots; To F—common dandelion flowers; To L—common dandelion leaves; To R—common dandelion roots; Tp F—red clover flowers; Ur L—nettle leaves; Ur R—nettle roots; Vo R—valerian roots.

**Table 12:** The presence of plant hormones in botanical extracts (μg∙mL^−1^).

Method	ABA	BA	GA_3_	IAA	JA	SA	Z
**Alv L**	0.84 ± 0.00	0.10 ± 0.00	87.48 ± 0.00	0.28 ± 0.00	ta	ta	0.04 ± 0.00
**Am Fr**	ta	0.03 ± 0.00	81.11 ± 0.00	0.81 ± 0.00	ta	ta	ta
**Arv H**	1.00 ± 0.00	0.08 ± 0.00	29.07 ± 0.00	ta	ta	0.02 ± 0.00	ta
**Bv R**	0.34 ± 0.00	0.23 ± 0.00	160.21 ± 0.00	0.91 ± 0.00	ta	ta	0.10 ± 0.00
**Co F**	0.35 ± 0.00	ta	185.71 ± 0.00	0.97 ± 0.00	0.03 ± 0.00	ta	ta
**Ea H**	ta	ta	168.30 ± 0.00	1.24 ± 0.00	ta	0.12 ± 0.00	ta
**Ep F**	1.23 ± 0.00	0.13 ± 0.00	319.23 ± 0.00	2.06 ± 0.00	ta	ta	ta
**Ep L**	0.11 ± 0.00	0.09 ± 0.00	87.90 ± 0.00	0.48 ± 0.00	ta	ta	ta
**Hp H**	1.00 ± 0.00	ta	72.19 ± 0.00	0.34 ± 0.00	ta	ta	0.09 ± 0.09
**Hr Fr**	ta	ta	66.91 ± 0.00	1.93 ± 0.00	0.05 ± 0.00	0.05 ± 0.00	ta
**Lc S**	ta	0.01 ± 0.00	101.80 ± 0.00	1.05 ± 0.00	ta	0.15 ± 0.00	ta
**Mc F**	1.50 ± 0.00	0.01 ± 0.00	125.69 ± 0.00	1.50 ± 0.00	ta	ta	ta
**Ob H**	1.07 ± 0.00	0.30 ± 0.00	94.80 ± 0.00	0.72 ± 0.00	ta	ta	0.05 ± 0.00
**Pm H**	0.70 ± 0.00	0.28 ± 0.00	343.92 ± 0.00	2.07 ± 0.00	ta	ta	ta
**Poa H**	ta	ta	162.40 ± 0.00	1.26 ± 0.00	0.04 ± 0.00	0.11 ± 0.00	ta
**Ps S**	ta	0.10 ± 0.00	87.57 ± 0.00	2.71 ± 0.00	ta	ta	ta
**Pta L**	ta	0.07 ± 0.00	56.33 ± 0.00	ta	ta	ta	ta
**Sg L**	0.20 ± 0.00	ta	359.85 ± 0.00	0.58 ± 0.00	ta	ta	ta
**So R**	0.15 ± 0.00	0.03 ± 0.00	144.98 ± 0.00	0.43 ± 0.00	ta	ta	0.20 ± 0.00
**To F**	0.49 ± 0.00	0.09 ± 0.00	134.40 ± 0.00	1.13 ± 0.00	ta	ta	0.21 ± 0.00
**To L**	0.64 ± 0.00	0.45 ± 0.00	88.62 ± 0.00	1.02 ± 0.00	ta	ta	0.17 ± 0.00
**To R**	0.85 ± 0.00	0.48 ± 0.00	325.19 ± 0.00	2.00 ± 0.00	ta	ta	0.05 ± 0.00
**Tp F**	1.36 ± 0.01	0.32 ± 0.00	76.90 ± 0.00	1.36 ± 0.01	0.01 ± 0.00	ta	ta
**Ur L**	ta	ta	324.83 ± 0.00	0.70 ± 0.00	ta	ta	ta
**Ur R**	ta	ta	359.47 ± 0.01	0.57 ± 0.00	ta	ta	ta
**Vo R**	0.30 ± 0.00	0.11 ± 0.00	85.55 ± 0.00	0.77 ± 0.00	ta	ta	ta

ta—trace amounts. Abbreviations: Alv L—aloe leaves; Am Fr—black chokeberry fruits; Arv H—common mugwort herb; Bv R—beetroot roots; Co F—common marigold flowers; Ea H—field horsetail herb; Ep F—purple coneflower flowers; Ep L—purple coneflower leaves; Hp H—St. John’s wort herb; Hr Fr—sea-buckthorn fruits; Lc S—red lentil seeds; Mc F—chamomile flowers; Ob H—basil herb; Pm H—broadleaf plantain herb; Poa H—common knotgrass herb; Ps S—pea seeds; Pta L—common bracken leaves; Sg L—giant goldenrod leaves; So R—comfrey roots; To F—common dandelion flowers; To L—common dandelion leaves; To R—common dandelion roots; Tp F—red clover flowers; Ur L—nettle leaves; Ur R—nettle roots; Vo R—valerian roots.

## Data Availability

Not applicable.

## Supplementary Materials

The following supporting information can be downloaded at: https://www.mdpi.com/article/10.3390/molecules28145572/s1, Table S1: The comparison of methods used to detect phenolic compounds (PC); Table S2: The comparison of methods used to detect tannins (TN); Table S3: The comparison of methods used to detect flavonoids (FD); Table S4: The comparison of methods used to detect anthocyanins (AC), and flavones (FL); Table S5: The comparison of methods used to detect quinones (QNO); Table S6: The comparison of methods used to detect glycosides; Table S7: The comparison of methods used to detect sugars.
