# Supplementary material for: Investigation of Chemical Constituents and Antioxidant Activity of Biologically Active Plant-Derived Natural Products
Source: Molecules. 2023 Jul 21;28(14):5572. doi: 10.3390/molecules28145572 (PMC10384900; doi:10.3390/molecules28145572)
Supplement: Supplementary file 1 [file molecules-28-05572-s001.zip › molecules-2478903-supplementary.pdf]

Table S1. The comparison of methods used to detect phenolic compounds (PC).

Method	Ferric chloride test	Lead acetate test	Zinc hydrochloride test	Shinoda test	Folin-Ciocalteu test
Extract	PC				mg·ml^{-1}
Alv L	-	+	-	+	0.36±0.00
Am Fr	+	-	-	-	1.13±0.02
Arv H	+	-	-/+	+	1.00±0.12
Bv R	-	+	+	-	0.53±0.02
Co F	+	-/+	+	-/+	0.75±0.00
Ea H	+	-/+	-/+	+	0.42±0.02
Ep F	+	-	+	-	3.17±0.03
Ep L	+	-	+	+	2.20±0.02
Hp H	+	-	-	-/+	1.47±0.05
Hr Fr	+	-	-	-	0.52±0.02
Lc S	-	-	-/+	-	0.13±0.01
Mc F	+	+	-/+	-/+	0.50±0.02
Ob H	+	+	-/+	+	1.44±0.03
Pm H	+	+	-/+	+	0.92±0.04
Poa H	+	-	-	+	0.36±0.00
Ps S	-	-	-	-	0.07±0.02
Pta L	+	-	-/+	+	3.11±0.03
Sg L	+	-	+	+	1.65±0.04
So R	+	-	-	-	0.88±0.02
To F	+	-	-/+	+	0.55±0.00
To L	+	-/+	-/+	+	1.14±0.02
To R	-	-/+	-	-/+	0.18±0.01
Tp F	+	-	+	-	0.82±0.04
Ur L	+	-	+	-/+	0.13±0.01
Ur R	-	+	+	-/+	0.09±0.01
Vo R	+	-/+	+	+	0.46±0.01
rpb+	0.46	-0.32	0.24	0.01	
rpb-	0.46	-0.19	0.15	0.24	

Table S2. The comparison of methods used to detect tannins (TN).

Method	Ferric chloride test	Lead acetate test	Gelatin test	Alkaline reagent test	Bromine water test	Potassium dichromate test	HCl test
Extract	TN	TN	TN	TN	TN	TN	TN
Alv L	-	+	-	-	-	-	-
Am Fr	+	-	-	-	-	-	-
Arv H	+	+	-	-	+	-	-
Bv R	-	+	-	-	-	-	-/+
Co F	+	+	-	-	-	-	-/+
Ea H	+	+	-	+	-	-	-
Ep F	+	-	-	-	-	-	-
Ep L	+	-	-	-	-	-	-
Hp H	+	-	+	-	-	-	-
Hr Fr	+	-	-	-	-	-	-
Lc S	-	-	+	+	-	-	-
Mc F	+	+	-	-	-	-	-
Ob H	+	+	-	-	-	-	-
Pm H	+	+	-	-	-	-	-
Poa H	+	+	-	-	-	-	-
Ps S	-	-	+	-	+	-	-
Pta L	+	+	-	+	-	-	-
Sg L	+	-	-	-	-	-	-
So R	+	-	-	-	-	-	+
To F	+	+	-	+	-	-	-
To L	+	+	-	-	-	-	-
To R	+	+	-	-	-	-	-
Tp F	+	-	-	-	-	-	-
Ur L	+	-	-	+	-	-	-
Ur R	-	+	-	-	+	-	-
Vo R	+	+	-	-	-	-	+

Table S3. The comparison of methods used to detect flavonoids (FD).

Method	Ferric chloride test	Lead acetate test	Zinc hydrochloride test	Shinoda test	Alkaline reagent test	Aluminium chloride test	Ammonium test	Ammonia and H_2SO_4 test
Extract	FD	FD	FD	FD	FD	FD	FD	FD
Alv L	-	-	-	-	-	-/+	-/+	-
Am Fr	-	-	+	+	-	-	-	-
Arv H	-	+	-	-	-	-/+	-	-
Bv R	-	-	-	+	-	-/+	-	-
Co F	-	+	-	-	-	-/+	-/+	+
Ea H	-	+	-	-	-	-/+	-/+	+
Ep F	-	-	-	+	-	-/+	-/+	-
Ep L	-	-	-	-	-	-/+	-/+	-
Hp H	-	-	-	-	-	-/+	-/+	-
Hr Fr	-	-	-	+	-	-/+	+	+
Lc S	-	-	-	+	-	+	-	-/+
Mc F	-	-	-	-	-	-/+	-/+	+
Ob H	-	-	-	-	-	-/+	-/+	-/+
Pm H	-	-	-	-	-	-/+	-/+	-/+
Poa H	-	+	-	-	-	-/+	+	-/+
Ps S	-	-	-	+	-	+	-	-
Pta L	-	+	-	-	-	-/+	-	-
Sg L	-	-	-	-	-	-/+	-/+	-/+
So R	-	-	-	-	-	-/+	-/+	-
To F	-	+	-	-	-	-/+	-/+	+
To L	-	+	-	-	-	-/+	-/+	-
To R	-	+	-	-	-	-/+	+	+
Tp F	-	-	-	+	-	-/+	-/+	+
Ur L	-	-	-	-	-	-/+	-	-/+
Ur R	-	-	-	-	-	-/+	-	-/+
Vo R	-	+	-	-	-	-/+	-	-

Table S4. The comparison of methods used to detect anthocyanins (AC), and flavones (FL).

Method	NaOH test	H_2SO_4 test	NaOH test	H_2SO_4 test
Extract	AC	AC	FL	FL
**Alv L**	-	+	-	+
**Am Fr**	-	+	-	+
**Arv H**	-	-/+	-	-/+
**Bv R**	-	-	-	-
**Co F**	-	-/+	-	-/+
**Ea H**	-	-/+	-/+	-/+
**Ep F**	-	-/+	-	-/+
**Ep L**	-	-/+	-/+	-/+
**Hp H**	-	+	-/+	+
**Hr Fr**	-	-	+	-
**Lc S**	-	-	-/+	-
**Mc F**	-	-	+	-
**Ob H**	-	-/+	-	-/+
**Pm H**	-	-/+	-/+	-/+
**Poa H**	-	+	+	+
**Ps S**	-	-	-/+	-
**Pta L**	-	+	-	+
**Sg L**	-	-/+	-	-/+
**So R**	-	-/+	-	-/+
**To F**	-	-/+	-	-/+
**To L**	-	-	-	-
**To R**	-	-	+	-
**Tp F**	-	+	-/+	+
**Ur L**	-	+	-/+	+
**Ur R**	-	-	-/+	-
**Vo R**	-	-	-	-

Table S5. The comparison of methods used to detect quinones (QNO).

Method	H_2SO_4 test	HCl test	Ammonia test
Extract	QNO	QNO	QNO
**Alv L**	-	-	-
**Am Fr**	+	-	-
**Arv H**	-	-	-
**Bv R**	-	-	-
**Co F**	-	-/+	-
**Ea H**	-	-	-
**Ep F**	-	-/+	-
**Ep L**	-	-	-
**Hp H**	-	-	-
**Hr Fr**	-	-	-
**Lc S**	-	-	-
**Mc F**	-	-	-
**Ob H**	-	-	-
**Pm H**	-	-	-
**Poa H**	-	-	-
**Ps S**	-	-	-
**Pta L**	-	-	-
**Sg L**	-	-	-
**So R**	-	-/+	-
**To F**	-	-	-
**To L**	-	-/+	-
**To R**	-	-	-
**Tp F**	-	-	-
**Ur L**	-	-	-
**Ur R**	-/+	-	-
**Vo R**	-	-	-

Table S6. The comparison of methods used to detect glycosides.

Method	Bromine water test	Baljet test	Keller-Killiani test	Borntrager's tests (1)	Borntrager's tests (2)	Molisch's test
Extract	GS	CGS	CGS	CYGS	GS	GS
Alv L	-	-	-	-	-	+
Am Fr	-	-	-	-	-	-
Arv H	-/+	-	-	-	-	+
Bv R	-	-	-/+	-	-	-
Co F	-	-	-/+	-	-	+
Ea H	-	-/+	-	-	-	+
Ep F	-	-	-/+	-	-	-
Ep L	-	-	-	-	-	-
Hp H	-	-	-/+	-	-	-
Hr Fr	-	-	-	-	-	-
Lc S	-	-	-	-	-	+
Mc F	-	-	-	-	-	+
Ob H	-	-/+	-	-	-	+
Pm H	-	-	-	-	-	-
Poa H	-	-	-	-	-	+
Ps S	-	-	-	-	-	+
Pta L	-	-/+	-	-	-	-
Sg L	-	-	-/+	-	-	+
So R	-	-	-/+	-	-	+
To F	-	-/+	-/+	-	-	-
To L	-	-	+	-	-	-
To R	-	-	-	-	-	-
Tp F	-	-	-	-	-	+
Ur L	-	-	+	-	-	+
Ur R	+	-	-	-	-	+
Vo R	-	-	+	-	-	-

Table S7. The comparison of methods used to detect sugars.

Method	Bromine water test	Borntrager's tests (2)	Molisch's test	Fehling's test	Benedict's test	Selwinoff's test	Barfoed's test
Extract	SG	SG	SG	SG	SG	SG	SG
**Alv L**	-	-	+	-	+	-	-
**Am Fr**	-	-	-	-	+	+	-
**Arv H**	-	-	+	-	+	-	+
**Bv R**	-	-	-	-	+	+	-
**Co F**	-	-	+	-	+	-	-
**Ea H**	-	-	+	-	+	-	-
**Ep F**	-	-	-	-	+	-	-
**Ep L**	-	-	-	-	+	-	-
**Hp H**	-	-	-	-	+	+	-
**Hr Fr**	-	-	-	-	+	-	-
**Lc S**	-	-	+	-	-	-	-
**Mc F**	-	-	+	-	+	-	-
**Ob H**	-	-	+	-	+	-	-
**Pm H**	-	-	-	-	+	-	-
**Poa H**	-	-	+	-	+	-	-
**Ps S**	-	-	+	-	-	-	-
**Pta L**	-	-	-	-	+	-	+
**Sg L**	-	-	+	-	+	-	-
**So R**	-	-	+	-	+	-	-
**To F**	-	-	-	-	+	-	-
**To L**	-	-	-	-	+	-	-
**To R**	-	-	-	-	+	-	+
**Tp F**	-	-	+	-	+	-	-
**Ur L**	-	-	+	-	+	-	-
**Ur R**	-	-	+	-	+	-	-
**Vo R**	-	-	-	-	+	-	-
